# Prognostic model based on telomere-related genes predicts the risk of oral squamous cell carcinoma

**DOI:** 10.1186/s12903-023-03157-x

**Published:** 2023-07-14

**Authors:** Kun Yue, Xue Yao

**Affiliations:** 1 Department of Stomatology, Weifang Hospital of Traditional Chinese Medicine, Weifang, 261000 Shandong China; 2 Department of Stomatology, Sunshine Union Hospital, 9000 Yingqian Road, High-tech Zone, Weifang, 261000 Shandong China

**Keywords:** Oral squamous cell carcinoma, Telomere-related genes, Prognostic model, Prognostic biomarkers, Function and pathway analysis, qPCR validation

## Abstract

**Background:**

This study investigated a potential prognostic model based on telomere-related genes (TRGs) for the clinical prediction of oral squamous cell carcinoma (OSCC).

**Methods:**

Gene expression data and associated clinical phenotypes were obtained from online databases. Differentially expressed (DE)-TRGs were identified between OSCC and normal samples, followed by protein-protein interaction and enrichment analyses. Subsequently, the prognostic genes explored based on the DE-TRGs and survival data were applied in the establishment of the current prognostic model, and an integrated analysis was performed between high- and low-risk groups using a prognostic model. The expression of certain prognostic genes identified in the present study was validated using qPCR analysis and/or western blot in OSCC cell lines and clinical samples.

**Results:**

169 DE-TRGs were identified between the OSCC samples and controls. DE-TRGs are mainly involved in functions such as hypoxia response and pathways such as the cell cycle. Eight TRGs (CCNB1, PDK4, PLOD2, RACGAP1, MET, PLK1, KPNA2, and CCNA2) associated with OSCC survival and prognosis were used to construct a prognostic model. qPCR analysis and western blot showed that most of the eight prognostic genes were consistent with the current bioinformatics results. Analysis of the high- and low-risk groups for OSCC determined by the prognostic model showed that the current prognostic model was reliable.

**Conclusions:**

A novel prognostic model for OSCC was constructed by TRGs. PLOD2 and APLK1 may participate in the progression of OSCC via responses to hypoxia and cell cycle pathways, respectively. TRGs, including KPNA2 and CCNA2, may serve as novel prognostic biomarkers for OSCC.

**Supplementary Information:**

The online version contains supplementary material available at 10.1186/s12903-023-03157-x.

## Background

Oral squamous cell carcinoma (OSCC) ranks as the 6th − 8th most common cancer globally [1]. Although numerous efforts have been made to understand the mechanisms of OSCC, such as gene expression [2], and to explore novel therapies [3], it still causes significant mortality worldwide, especially in China [4]. Screening for risk factors and prognostic targets related to OSCC has been a major focus of previous studies [5]. However, the intractability and high morbidity rate of OSCC has caused severe personal distress and social and financial burden.

Over recent years, genome-wide association studies of increasing size have identified that telomere variants contribute to the progression of human disease [6]. During this process, abnormal nuclear morphologies induced by telomere dysfunction might be an important reason [7].Telomere-related genes (TRGs) play a vital role in protecting chromosomal structure [8]. In an experimental model, telomere length and genetic variants in TRGs are significantly different in patients with atrophic age-related macular degeneration when compared with healthy controls, indicating the important role of TRGs in human disease [9]. As important factors involved in the maintenance of chromosome structures, TRGs have been confirmed to participate in the occurrence and development of tumors [10]. Telomere dysfunction can lead to biological dysfunction as well as tumorigenesis in human diseases such as OSCC [11]. It has been demonstrated that that telomere dysfunction can lead to perturbation in various cell signaling pathways during the progression of neck squamous cell carcinoma [12]. The prognostic significance of telomere genes has been revealed in breast carcinoma [13]. Bulter et al. indicated that differentially expressed TRGs (DE-TRGs), such as the telomere repeat binding factor 1 (TRF1) cloud, could be used as prognostic indicators in human breast cancer because they participate in telomere maintenance [14]. Since TRGs are essential structures for maintaining chromosomal stability, their variation contribute to the cancer risk and clinical outcome [13]. Based on that, the prognostic model established based on TRGs has more advantages in predicting the prognosis of cancer patients compared with other models [15].Therefore, prognostic methods based on TRGs are increasingly attracting the attention of researchers and clinicians for application in clinical interventions in human cancers [16]. Chang et al. indicated that TRGs mutations play a vital role in the pathogenesis and progression of oral cavity squamous cell carcinoma, which further indicating the importance of prognostic model based on TRGs in squamous cell carcinoma [17]. However, whether a TRG-based prognostic model can be constructed and successfully used for the clinical prediction of OSCC remains unknown. Therefore, trying to establish the prognostic model of OSCC based on TRGs is of great significance for the clinical research of OSCC.

In the present study, OSCC-associated DE-TRGs were identified between OSCC samples and normal controls based on the TCGA dataset and the TelNet database. Subsequently, the prognostic genes identified based on the DE-TRGs and survival data were used to construct a prognostic model, and the associations between the prognostic model and survival were revealed. Finally, based on OSCC cell lines, the expression of certain prognostic genes identified in the present study was validated by qPCR analysis. The aim of the present study was to develop an applicable prognostic model and reliable biomarkers for OSCC.

## Methods

### Microarray data and pre-processing

RNA-seq data (log2(fpkm + 1)) of GDC TCGA HNSC were obtained from the UCSC Xene database (https://xenabrowser.net/) [18]. Samples from the tongue, mouth, gum, lip, cheek mucosa, and palate were selected based on the source of the diagnosed tissues or organs. Subsequently, samples with “-01A” in the sample tissue number were enrolled as OSCC samples (OSCC group), while samples with “-11A” were enrolled as normal samples (N group). Finally, a total of 255 OSCC samples (with survival and clinical information provided in online database) and 19 normal samples were used for further investigation. All the data obtained in the present study were updated in September 2022. According to the downloaded gene annotation file of the relevant version of GENCODE V22, genes with annotation information of “protein_coding” were reserved for the present analysis.

Moreover, the microarray dataset GSE42743 in the Gene Expression Omnibus (GEO) database (http://www.ncbi.nlm.nih.gov/geo/) [19] was acquired for external validation, and it contained 71 OSCC tumor samples with clinical survival and prognosis information. The data were generated using a GPL570 Affymetrix Human Genome U133 Plus 2.0 Array. Probes that did not correspond to the gene symbols were excluded based on the probe expression matrix and annotation file. If multiple probes corresponded to the same gene, the average value was used as the gene expression value.

### DE-TRG investigation

The differentially expressed genes (DEGs) between the OSCC and N groups from the TCGA-OSCC dataset were explored using the classic Bayesian method in the limma package (version3.10.3) [20]. Briefly, significance analyses for the expression of all genes were performed based on the log fold change (FC) and P value. The selection threshold for DEGs was Benjamini & Hochberg (BH) adj. P value < 0.05, and |logFC| > 1. Subsequently, TRGs obtained from the TelNet database were integrated with the DEGs to obtain DE-TRGs. Finally, the DE-TRGs were included in the subsequent analysis.

### Correlation analysis and PPI network investigation

The Pearson correlation coefficient method was used to reveal the correlation coefficient and significant P value between the two DE-TRGS. Afterward, according to the STING database (version:11.0, species: Homo sapiens) [21], the protein interactions were extracted, and the relationships among proteins were revealed based on the score = 0.9, followed by network establishment using Cytoscape software (version:3.6.1) [22]. The connection degree of node in the network was further investigated based on the network topology in the CytoNCA software (Version 2.1.6, parameters: without weight) [23].

### Enrichment analysis for DE-TRGs

GO function and KEGG [24–26] pathway analyses were performed for the DE-TRGs using DAVID software (version 6.8) [27]. The GO functions included biological process (BP), cellular components (CC), and molecular function (MF). The P < 0.05 and count (the number of genes enriched in certain item) ≥ 2 were used as the cut-off values.

### Prognostic gene investigation

Using the survival package (version: 2.41-1) [28] in R software, univariate Cox regression analysis was performed to reveal DE-TRGs related to overall survival and prognosis based on the expression value of DE-TRGs and sample prognostic information.

### Prognostic model establishment

Based on the LASSO Cox regression in R (version:3.6.1) [29], the optimal gene set was investigated from the prognostic DE-TRGs in the TCGA-OSCC training dataset that was analyzed with 20 fold (nfold = 20s) cross-validation analysis provided by the glment package (version:2.0–18). Finally, the following Riskscore (RS) model was established:

$${\rm{RS}}\,{\rm{ = }}\,\sum {{\rm{\beta }}_{{\rm{gene}}}}{\rm{ \times Exp}}{\,_{{\rm{gene}}}}$$

In the formula, β_gene_ represents Cox regression prognostic coefficient for the target gene. Exp _genes_ represents the expression levels of TRGs in each sample.

### Prognostic model validation

According to the prognostic coefficients of the signature genes, the median RS of the two sample sets, GSE14520 and TCGA-OSCC, was calculated, and the samples were divided into high-risk and low-risk groups. Kaplan-Meier (KM) curves were used to analyze survival in the high- and low-risk groups. Finally, based on the survival information of the samples, ROC curves of the 1-, 3-, and 5-year survival rates were obtained for TCGA-OSCC and GSE42743.

### Independent analysis of prognostic model

Univariate and multivariate Cox regression analyses based on age, neoplasm_histologic_grade, pathologic_N, pathologic_T, gender, tumor_stage, and risk score were performed for the independent analysis of the model. The log-rank *P* < 0.05 was used as the cutoff value for significant correlations. Clinical factors associated with independent prognoses were investigated using nomograms. Meanwhile, verification of the of nomogram effectiveness was performed based on correction curve.

### Immune cell infiltration analysis between two risk groups

The gene set used to label each type of infiltrating immune cell was obtained from a study by Charoentong et al. [30]. Based on the ssGSEA algorithm and Gene Set Variation Analysis (GSVA) in R [31], the enrichment fraction of each immune cell in different samples of TCGA-OSCC was calculated to represent the relative abundance of each infiltrating cell in each sample. Finally, the Wilcoxon signed-rank test was used to reveal the differences in immune cells between the two groups.

### Expression analysis of immune checkpoint gene and key immunosuppressive genes between two risk groups

Based on the TCGA-OSCC samples, the expression of immune checkpoint genes, including PDCD1 (PD-1), CD274 (PD-L1), CTLA4, IDO1, CD96, TIGIT, LAG3, and PVR were extracted, and the difference in expression between the low-risk and high-risk groups was revealed using an inter-group T test. Furthermore, based on TCGA-OSCC samples, the expression levels of key immunosuppressive genes, including IL10, TGFB1, FOXP3, IL6, and FAP, were extracted, followed by the investigation of expression differences between the two groups using the inter-group T test.

### KEGG pathway and HALLMARK gene set enrichment analysis between two risk groups

Based on the c2.cp.kegg.v7.4. symbols.gmt and h.all.v7.4.symbols.gmt enrichment background in the MSigDB v7.1 database [32], the enrichment scores of each KEGG pathway and HALLMARK gene set in each TCGA-OSCC sample were calculated and sorted using the GSVA algorithm in R [31] with BH adjusted P < 0.05.

### Distribution analysis of clinical characteristics between two risk groups

According to the clinical characteristics of each group in TCGA-OSCC, Wilcoxon’s signed-rank test was used to reveal the difference in risk score between the high- and low-risk groups. The results were visualized using a heatmap.

### Drug sensitivity analysis between two risk groups

To reveal differences in sensitivity (IC50 value difference) for common chemotherapy drugs in the two groups, a ridge regression model was constructed according to GDSC cell lines and the TCGA-OSCC gene expression profile by using the pRRophic algorithm [33], so as to represent the drug sensitivity. Finally, the Wilcoxon signed-rank test was used to determine the difference in IC50 values of each drug between the two groups.

### Cell culture

A total of three human OSCC cell lines, including CAL-27, SCC-25, and SCC-9 and normal oral keratinocytes (NOK) were purchased from the Cell Bank of Chinese Academy of Sciences (Shanghai, China). The mixture of Roswell Park Memorial Institute (RPMI) 1640 medium (Hyclone, USA) and fetal bovine serum (Hyclone, USA), with 10% fetal bovine serum were used for current cell culture. Cells were maintained in an incubator at 37˚C with 5% CO_2_.

### Clinical samples

Five paired mucosa tissue samples (tumor and adjacent normal tissues) were collected from SOCC patients (3 males and 2 females, mean age: 59.2 ± 5.3 year) under surgery in our hospital. The included patients were presented with no chronic disorder and had not received any treatment prior to experiments. Informed consent had been signed and this study was approved by the Ethics Committee of our hospital.

### Real time PCR assay

Total RNAs were extracted from cells and tissues by using TRIZOL reagent (Invitrogen, USA) and reversely transcribed using the ^RevertAidTM^ First Strand cDNA Synthesis Kit (Thermo Fisher Scientific) in accordance with the manufacturer’s instructions. PCR was performed on an ABI7500 Fast real-time PCR instrument (Applied Biosystems, USA). In the detection of PLOD2 expression (F:5′- GCGTTCTCTTCGTCCTCATC − 3′; R:5′- GTGTGAGTCTCCCAGGATGC − 3′), MET expression (F:5′- CCCCACCCTTTGTTCAG − 3′; R:5′- TCAGCCTTGTCCCTCCT − 3′), PLK1 expression (F:5′- ATGAGTGCTGCAGTGACTGC − 3′; R:5′- TTAGGAGGCCTTGAGACGGT − 3′), CCNA2 expression (F:5′- GCCAAGCTAACCAAAGCTC − 3′; R:5′- CATAAAGAGGCTACCATAA − 3′), KPNA2 expression (F:5′- ATTGCAGGTGATGGCTCAGT − 3′; R:5′- CTGCTCAACAGCATCTATCG − 3′), and PDK4 expression (F:5′- GGAGCATTTCTCGCGCTACA − 3′; R:5′- ACAGGCAATTCTTGTCGCAAA − 3′). GAPDH was used as an internal control (F:5′- CAAGGTCATCCATGACAACTTCG − 3′; R:5′- GTCCACCACCCTGTTGCTGTAG − 3′). The PCR program included 95 °C for 5 min, 35 cycles of 95 °C for 30 s and 52 °C for 30s. The relative expression was calculated using the 2 ^−ΔΔCt^ method [34].

### Western blot

The proteins of each sample were isolated by centrifugation after tissue specimens were lysed with the use of PIRA solution. Then, the qualified protein samples were separated by 10% SDS-PAGE electrophoresis and transferred on PVDF membranes. Immune staining was performed with the primary antibodies against mouse PLOD2 (1:1000, Invitrogen), rabbit MET (1:1000, cell signaling technology), rabbit PLK1 (1:1000, cell signaling technology), rabbit CCNA2 (1:1000, cell signaling technology), rabbit KPNA2 (1:1000, cell signaling technology) and mouse PDK4 (1:1000, cell signaling technology), followed by the goat anti-rabbit or anti-mouse IgG antibodies (1:2000, cell signaling technology). The proteins were visualized by ECL system and analyzed by Image J software against DAPDH.

### Statistical analysis

GraphPad prism 5 (GraphPad Software, San Diego, CA, USA) was used to generate graph. The results were calculated as mean ± standard deviation (SD) values. The significance of differences between group pairs was calculated using Student’s t-test. P-values of < 0.05 indicated statistical significance.

## Results

### DE-TRG investigation

**Fig. 1:**
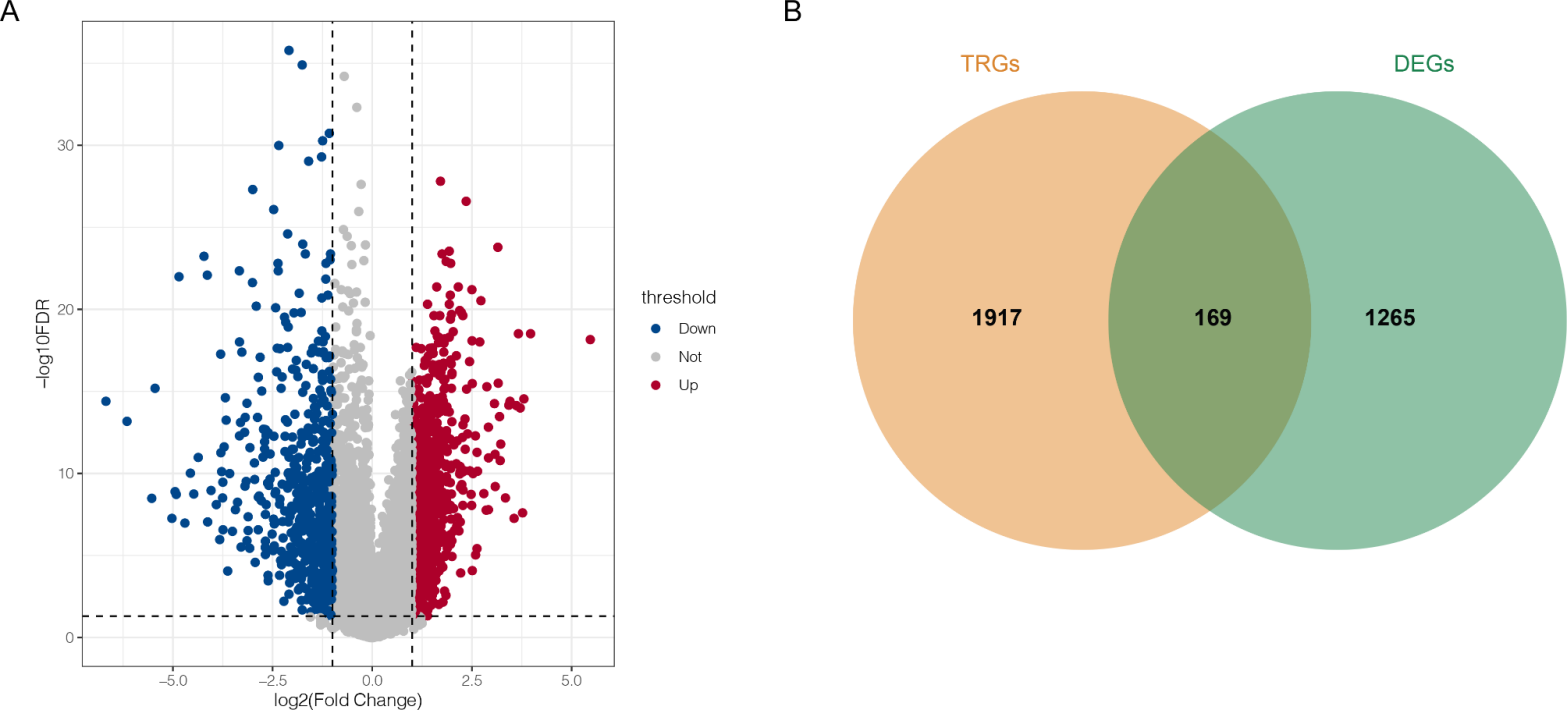
The differentially expressed genes (DEGs) between oral squamous cell carcinoma (OSCC) samples and normal samples. **A**, the differentially expressed genes between OSCC samples and normal samples revealed by volcano plot; red circle represented up-regulated gene, while blue circle represented down-regulated gene; the grey circle represented gene without significant difference between two groups. **B**, the VENN plot revealed the differentially expressed telomere related genes (DE-TRGs) between DEGs and telomere related genes (TRGs).

After annotation, 19,710 gene expression values were obtained from 255 OSCC samples (OSCC group) and 19 normal samples (N group) in the TCGA database. Differential expression analysis revealed 855 upregulated and 579 downregulated genes in the OSCC and N groups. As shown in Fig. 1A, the up-regulated TRGs (marked red) and down-regulated TRGs (marked blue) were separated by groups. Then, a total of 2086 TRGs were downloaded from the TelNet database, followed by 169 DE-TRGs that were explored by matching them with DEGs (Fig. 1B).

#### Protein–protein interaction network investigation

**Fig. 2:**
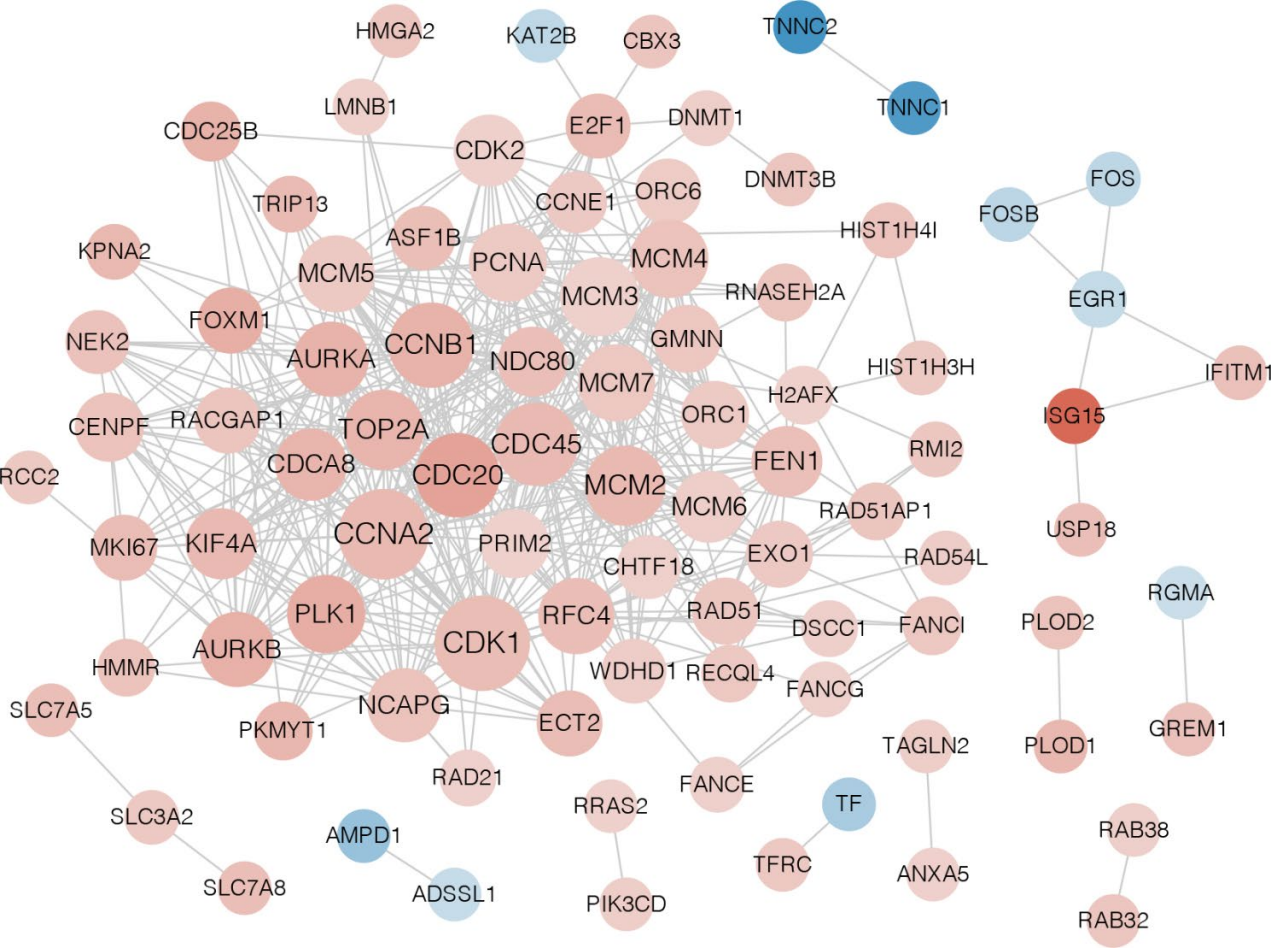
The protein-protein interaction network constructed by DE-TRGs. The red nodes represented up-regulated genes; the blue nodes represented down-regulated genes. The line between two nodes represented interaction. The darker the color, the greater the difference; the larger the node, the greater the connectivity

Interactions among the DE-TRGs were investigated using protein–protein interaction (PPI) network analysis. The results showed 458 interactions and 90 nodes (10 downregulated and 80 upregulated DE-TRGs) were used to construct the current PPI network. Detailed information is shown in Fig. 2.

### Enrichment analysis

**Fig. 3:**
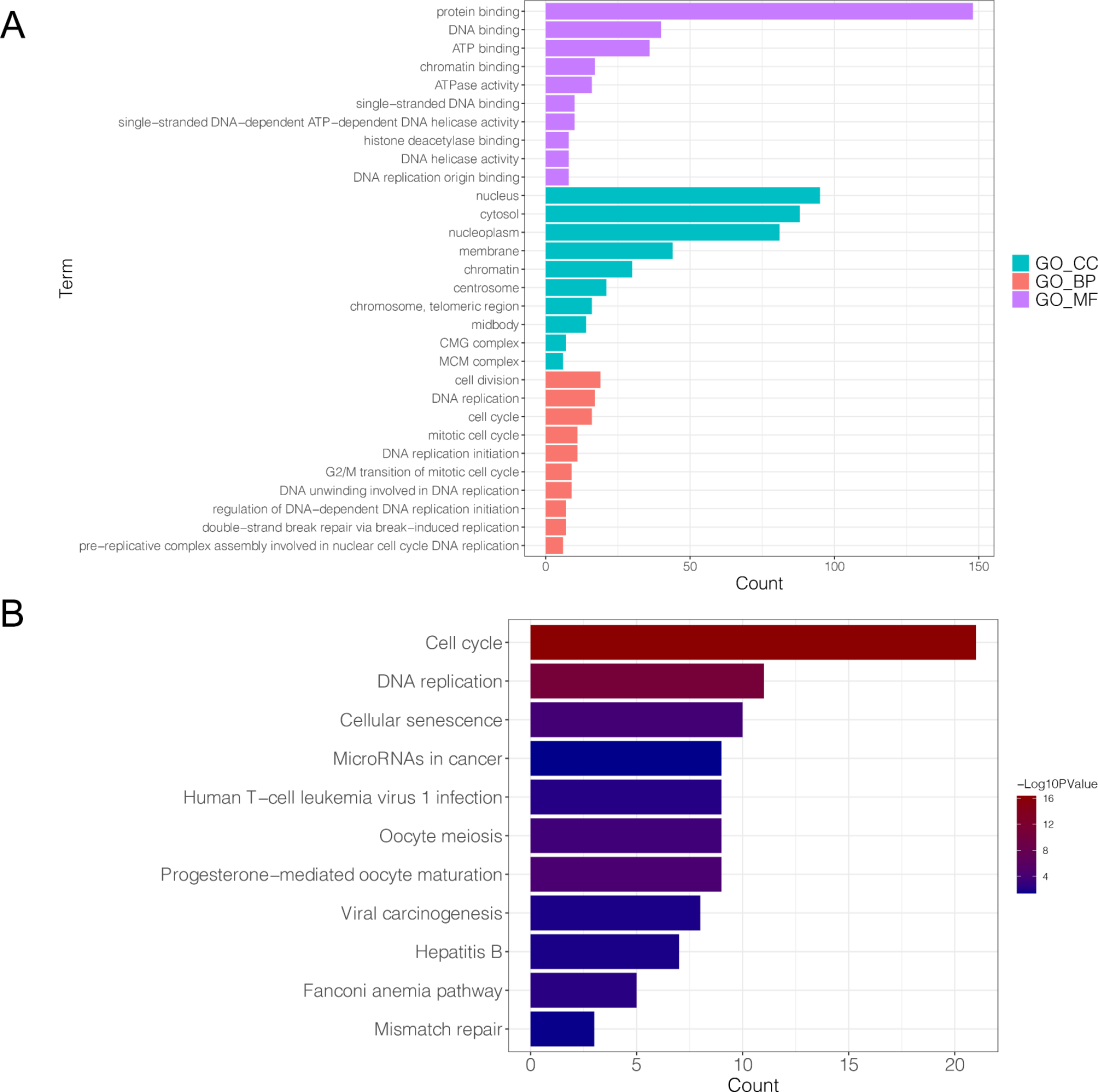
The result of enrichment analysis in current study. **A**, the Top5 GO-BP, GO-CC and GO-MF functions assembled by DE-TRGs; the X-axis represented different GO items, while the Y-axis represented the value of count (the number of genes in certain item). **B**, the pathways enriched by DE-TRGs; the X-axis represented different KEGG items, while the Y-axis represented the value of count; the deeper the color, the significant the P value

The results of enrichment analysis showed that the genes were mainly assembled into 209 GO functions, including response to hypoxia (BP, GO: 0001666; Genes: PLOD2, PLOD1, EGR1, etc.), nucleus (CC, GO:0005634; Genes: TOP2A, FEN1, CLIC3, etc.), and protein binding (MF, GO:0005515; Genes: IFITM1, IL1RN, TFRC, etc.). The top five results for GO-BP, GO-CC, and GO-MF are shown in Fig. 3A. The genes were mainly enriched in 12 pathways, including cell cycle (hsa: 04110; Genes: PLK1, PKMYT1, CCNA2, etc.) and DNA replication (hsa:03030; Genes: PRIM2, FEN1, RFC4, etc.) (Fig. 3B). The detail information of current enrichment analysis was listed in Supplementary Table 1.

### Prognostic gene exploration and correlation analysis

**Fig. 4:**
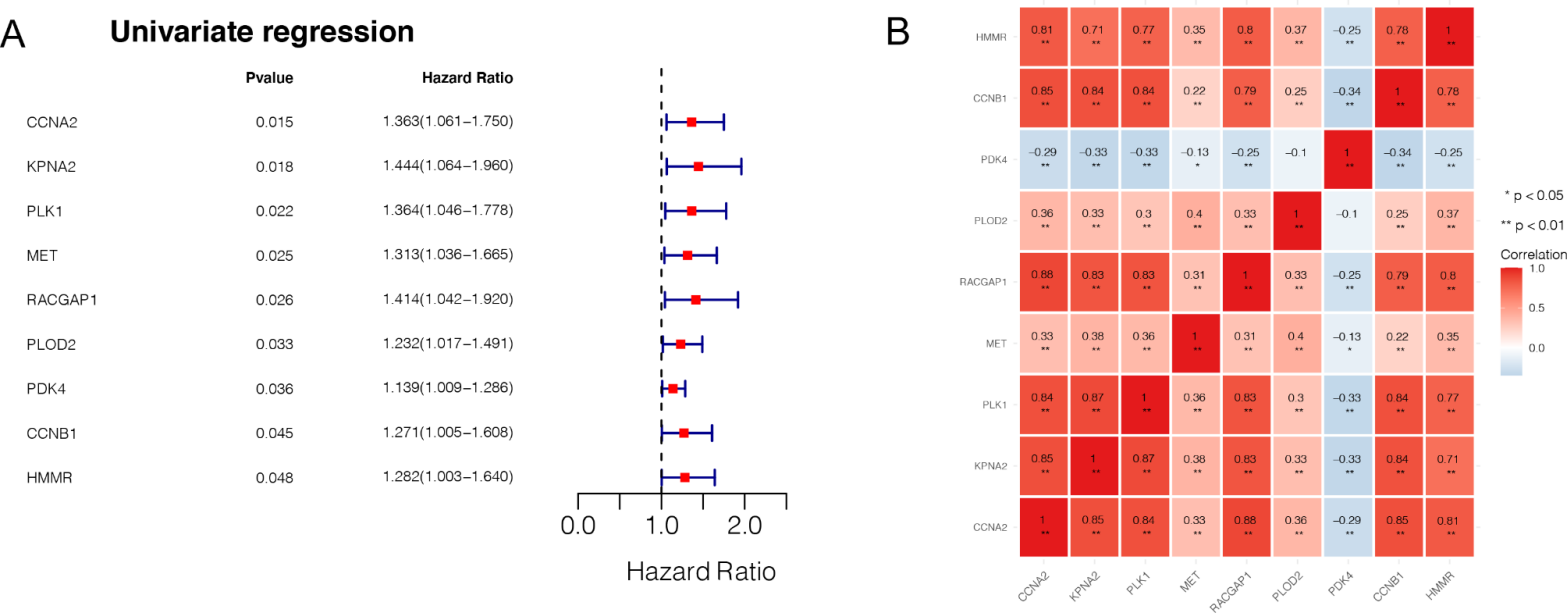
The prognostic genes revealed based on DE-TRGs. **A**, the forest map of for all prognostic genes reveled based on univariate Cox regression analysis. **B**, Pearson correlation coefficient method was used to calculate the correlation between two prognostic genes; the redder the color, the more significant the correlation

Univariate Cox regression analysis of DE-TRGs revealed nine genes, including HMMR, CCNB1, PDK4, PLOD2, RACGAP1, MET, PLK1, KPNA2, and CCNA2, which were significantly associated with the overall survival and prognosis of OSCC (P < 0.05, Fig. 4A). The results of correlation analysis of the nine prognostic genes are shown in Fig. 4B.

### Prognostic model construction and validation

**Fig. 5:**
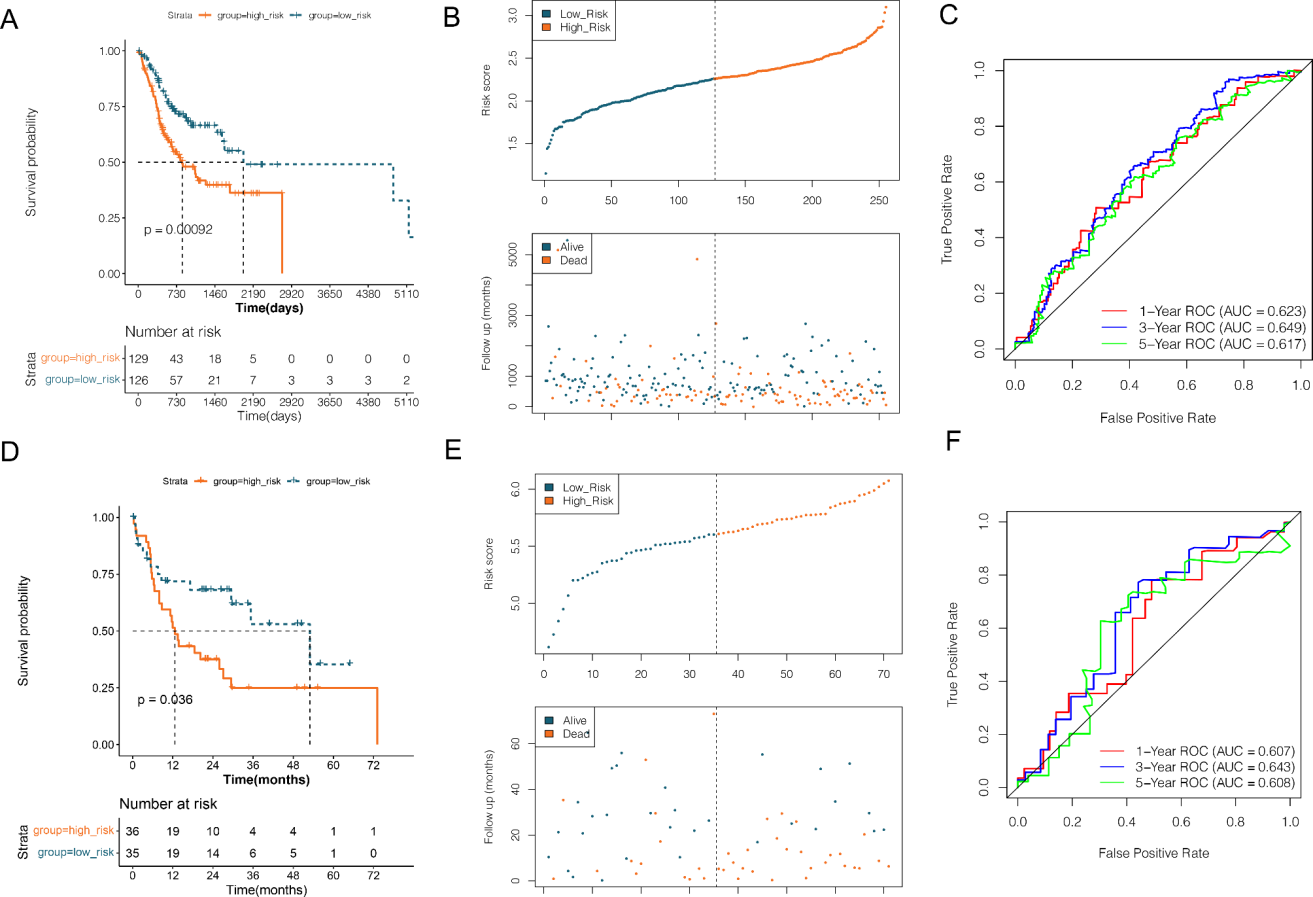
The prognostic model established in current study. **A**, the Kaplan Meier survival curve based on Riskscore model in TCGA-OSCC training dataset: the blue dot represented sample in high-risk group, while orange dot represented sample in low-risk group. **B**, survival distribution of samples from different risk groups in TCGA-OSCC training dataset: the blue dot represented sample in high-risk group, while orange dot represented sample in low-risk group. **C**, the ROC curve of 1-year, 3-year and 5-year survival based on samples in TCGA-OSCC training dataset. **D**, the Kaplan Meier survival curve based on Riskscore model in GSE42743 dataset: the blue dot represented sample in high-risk group, while orange dot represented sample in low-risk group. **E**, survival distribution of samples from different risk groups in GSE42743 dataset: the blue dot represented sample in high-risk group, while orange dot represented sample in low-risk group. **F**, the ROC curve of 1-year, 3-year and 5-year survival based on samples in GSE42743 dataset

Based on the 9 DE-TRGs that were significantly related to survival and prognosis obtained above, combined with their expression values in the TCGA-OSCC samples in the training set, the survival time and survival status of the samples, totally 8 optimized TRGs combinations were selected by using LASSO Cox expression algorithms (Supplementary Fig. 1). Based on eight optimal genes, including CCNB1, PDK4, PLOD2, RACGAP1, MET, PLK1, KPNA2, and CCNA2, in both TCGA-OSCC (Fig. 5A-C) and GSE42743 datasets (Fig. 5D-F), a TRGs associated prognostic model was constructed based on the gene expression level in TCGA and GSE42743 dataset. The samples were assigned to two risk groups based on the median risk score. Compared with the low-risk group, survival in the high-risk group was worse (Fig. 5B and D). ROC curves for 1-year, 3-year, and 5-year survival in TCGA-OSCC and GSE42743 are shown in Fig. 5 C and 5 F, respectively.

### The independent analysis for prognostic model

**Fig. 6:**
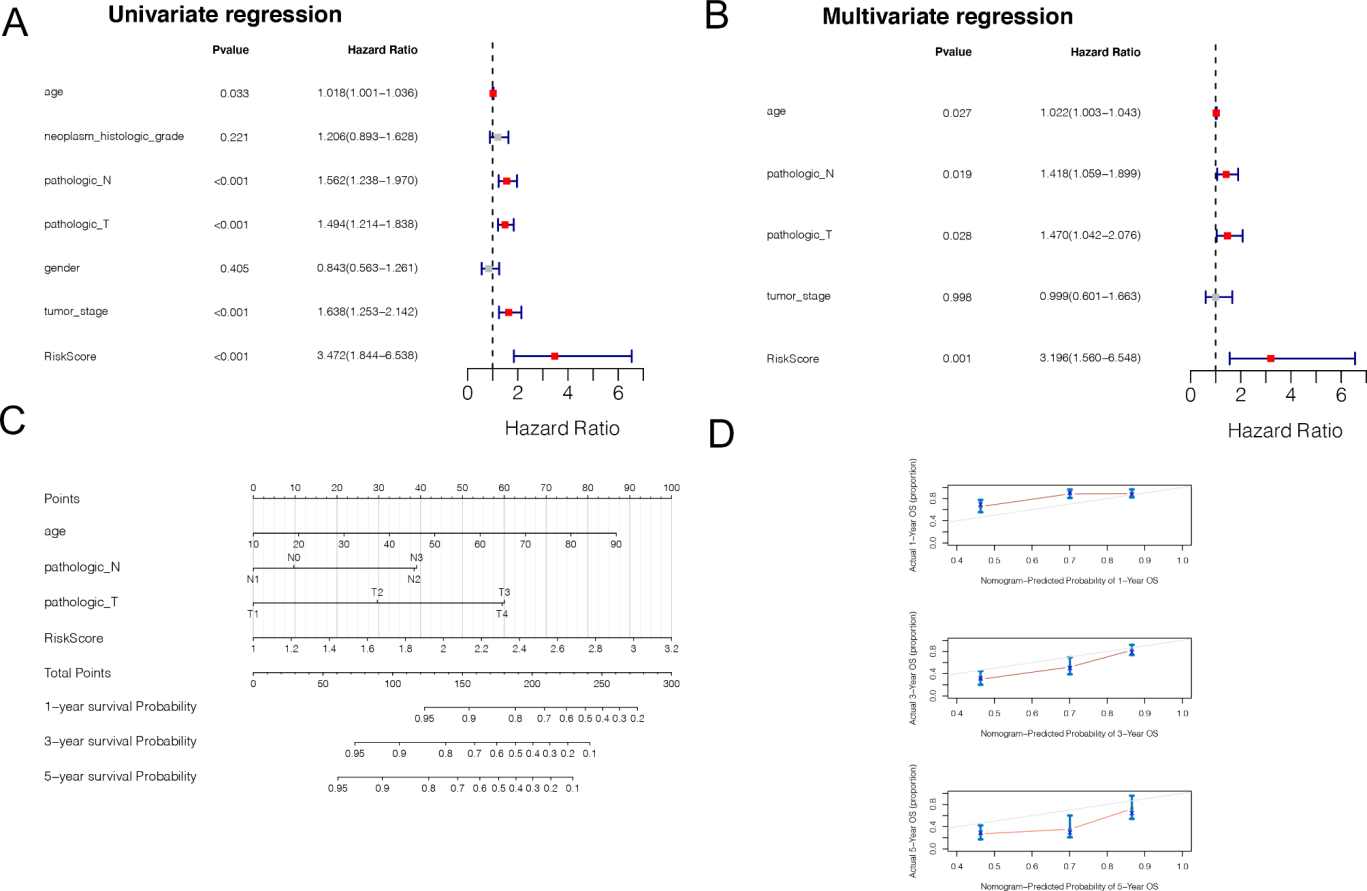
The Cox regression for the clinical risk factor of OSCC. **A**, the univariate regression for clinical factors (age, neoplasm_histologic_grade, pathologic_N, pathologic_T, gender, tumor_stage and RiskScore) and prognosis. **B**, the multivariate regression revealed the independent factors for OSCC. **C**, The predictive nomogram for risk factors in OSCC. **D**, the Nomogram predicted probability of 1-year, 3-year, and 5-year overall survival

According to the method section, the Cox regression analysis were performed on age, neoplam_ histologic_ grade, pathologic_ N, pathologic_ T, gender, tumor_ Stage and RiskScore respectively, followed by the multivariate cox regression analysis with P < 0.05. The result showed that the age, pathologic_N, pathologic_T, and risk score were found to be associated with prognosis (Fig. 6A). Multivariate Cox regression analysis was used to screen independent prognostic factors (Fig. 6B). The results of the nomogram-associated analysis showed that the factors were significantly correlated with prognosis (Fig. 6C-D).

### Immune cell infiltration, immune checkpoint gene expression, and immunosuppressive gene expression analysis between two risk groups

**Fig. 7:**
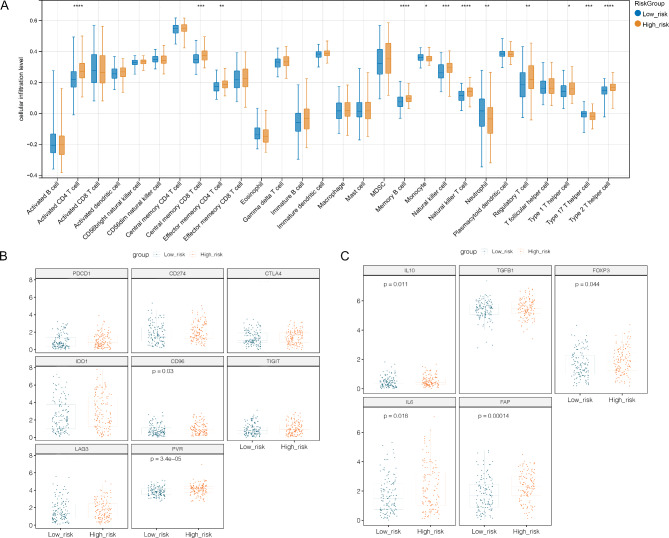
The immune associated analysis between high-risk group and low-risk group determined by prognostic model. **A**, box diagram revealed the result of immune cell infiltration between two groups: X-axis represented different immune cells, while the Y-axis represented cellular infiltration level. **B**, the box diagram for immune checkpoint genes expression between high-risk group and low-risk group. **C**, the box diagram of immunosuppressive genes expression between high-risk group and low-risk group

To compare the infiltration of immune cells between the two groups in the training set, the infiltration abundance of 28 types of immune cells was investigated based on ssGSEA algorithm. The results showed that 12 types of immune cells including activated CD4 T cell, Central memory CD4 T cell, Effector memory CD4 T cell, Memory B cell, Monocyte, Natural killer T cell, Neutrophil, Regulatory T cell, Type 1 T helper cell, Type 17 T helper cell and Type 2 T helper cell were significantly different between two groups (Fig. 7A). The expression levels of eight immune checkpoint genes, including PDCD1 (PD-1), CD274 (PD-L1), CTLA4, IDO1, CD96, TIGIT, LAG3 and PVR were compared between the two groups. The results showed that the expression of two immune checkpoint genes, CD96 and PVR, was significantly different between the two groups (Fig. 7B). Furthermore, immunosuppressive gene expression analysis showed that the levels of IL10, FOXP3, IL6, and FAP were significantly different between the two groups (Fig. 7C).

### KEGG pathway and GSEA analysis between two risk groups

The KEGG pathway and HALLMARK GSEA enrichment analysis was performed on genes between high risk group and low risk group. The results showed that 34 upregulated pathways and one downregulated pathway were significantly different between the groups in the current analysis. Moreover, 25 HALLMARK gene sets were significantly different between the groups. According to the NES ranking (the greater the absolute value of NES, the more obvious the enrichment), the top six pathways and gene sets are shown in Supplementary Fig. 2A–B.

### Clinical characteristics and drug sensitivity between two risk groups

**Fig. 8:**
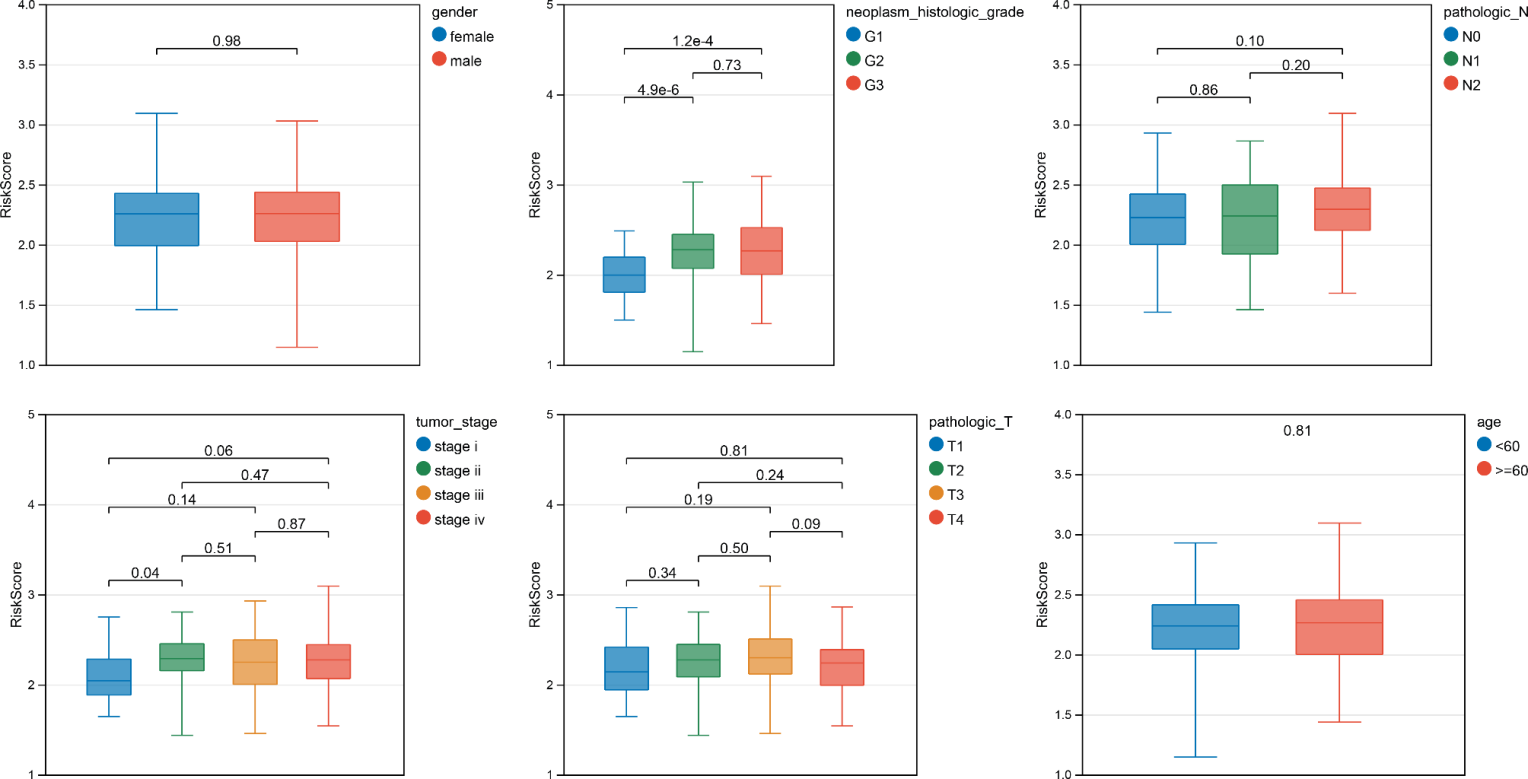
The result of clinical characteristics and drug sensitivity between high-risk group and low-risk group. The box diagram of Riskscore among different clinical groups: the number in the top represented the P value

According to the clinical characteristics of the two risk groups in TCGA-OSCC, the Wilcoxon test was used to investigate the difference in risk scores between the two groups. The results showed that only the neoplasm histological grade was significantly different (P < 0.05) between the two groups (Fig. 8). A heatmap of the eight prognostic genes including CCNA2, PLK1, MET, RACGAP1, PDK4, CCNB`, KPNA2 and PLOD2 among the different clinical groups is shown in Supplementary Fig. 3. Furthermore, a difference in IC50 values for sensitivity to common chemotherapeutic drugs between the two groups was revealed. The results showed that the IC50 values of 67 drugs, including Lapatinib, Pyrimethamine, and Gemcitabine, were significantly different between the two risk groups. For example, the IC50 values of Lapatinib in low-risk group was significantly lower than that in high-risk group, indicating a high tolerance to the Lapatinib in patients from low-risk group. Meanwhile, there was a good effective of Pyrimethamine and Gemcitabine in patients from high-risk group than that in low-risk group.

### Prognostic gene expression in cell lines

**Fig. 9:**
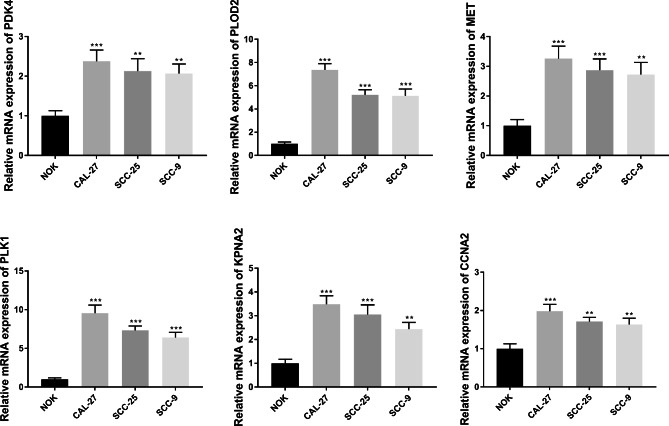
The results of qRT-PCR validation analysis for 6 TRGs in prognostic model. The X-axis represented different cells, while the Y-axis represented the relative mRNA expression of different TRGs. NOK, normal oral cells as control; CAL-27, SCC-25 and SCC-9, three different OSCC cell lines; **, P < 0.05 when compared with NOK; ***, P < 0.01 when compared with NOK.

To further investigate the expression of prognostic genes (PLOD2, MET, PLK1, CCNA2, KPNA2, and PDK4) revealed in the current study, verification studies were performed in SOCC cells. The expression of six prognostic genes (PLOD2, MET, PLK1, CCNA2, KPNA2, and PDK4) in the three cell lines and one normal oral cell line was investigated using qPCR. The results showed that the expression of all six prognostic genes in the three cell lines (CAL-27, SCC-25, and SCC-9) was significantly higher than that in NOK cells (all P < 0.05). The expression of the six prognostic genes in the verification analysis was consistent with the results of our current bioinformatics study, indicating a reliable result for this study. A bar chart of six relative gene expression of six genes in cell lines and normal oral cells is shown in Fig. 9.

### Validation in clinical samples

**Fig. 10:**
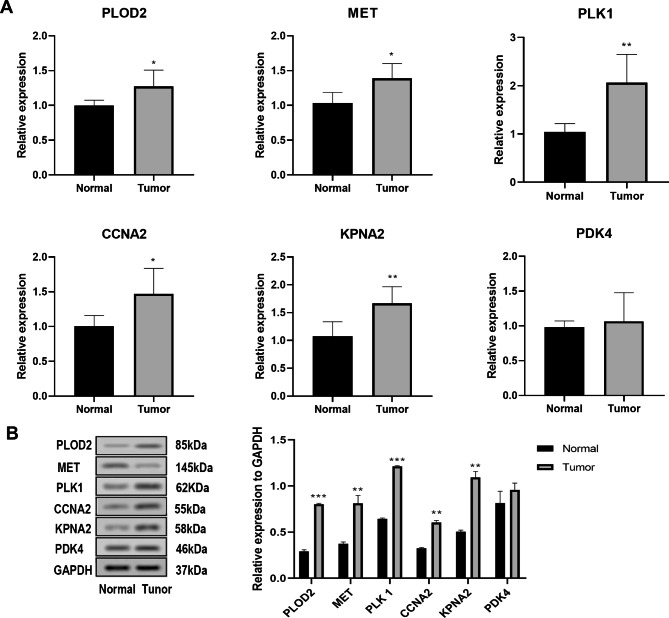
Validation analysis of 6 TRGs in clinical samples. **A**, qRT-PCR analysis. **B**, western blot analysis. *P < 0.05, **P < 0.01, ***P < 0.001, compared with normal ones

The differential expression of the prognostic genes was verified in SOCC tumor tissues at mRNA and protein level. qRT-PCR analysis determined that the expression of PLOD2, MET, PLK1, CCNA2, and KPNA2 were significantly upregulated in tumor tissues relative to the normal ones (all p < 0.05, Fig. 10A). Consistent results were observed in western blot (Fig. 10B). Notably, no significant difference was observed in the expression of PDK4 in tumor tissues and normal counterparts, which might be resulted from the small sample size. Thus, further experiments with large sample size were warranted.

## Discussion

OSCC is associated with high mortality rates globally. Although telomere-associated genetic variants have been shown to be associated with the risk and survival of OSCC [35], prognostic recognition of this disease remains limited. In the present study, eight upregulated TRGs (CCNB1, PDK4, PLOD2, RACGAP1, MET, PLK1, KPNA2, and CCNA2) were identified as prognostic genes for OSCC. Validation analysis showed that the expression of most prognostic genes in OSCC cells and clinical tissues was consistent with the bioinformatics results of the present study. The analysis of the high- and low-risk groups of OSCC determined by the prognostic model showed reliable results in the present study.

PLOD2 (Procollagen-Lysine,2-Oxoglutarate 5-Dioxygenase 2) is overexpressed in various types of tumors and plays an important role in tumorigenesis [36]. A previous study showed that PLOD2 mRNA and its associated proteins were significantly increased in cancer cells compared to those in normal controls [37]. Sun et al. reported that PLOD2 is an undesirable prognostic biomarker for patients since it influences OSCC metastasis through the epithelial–mesenchymal transformation (EMT) pathway [38]. By inducing EMT, hypoxia participates in OSCC tumor progression [39]. As a partially invasive cancer, OSCC is characterized by severe hypoxia and is less sensitive to chemotherapy [40]. It has been demonstrated that PLOD2 induced under hypoxia is involved in drug resistance and poor prognosis in human cancers [41]. In addition, PLK1 (Polo-like kinase 1) overexpression has been shown to occur in a wide range of tumors including OSCC [42]. A previous study indicated that PLK1 overexpression plays a critical role in either the occurrence or the progression of OSCC and can be used as a novel biomarker for OSCC [43]. Furthermore, Kahl et al. demonstrated that PLK1 is a cell cycle-related gene associated with a poor prognosis in human cancer [44]. PLK1 takes part in the regulation of tumor cell via G2/M DNA damage checkpoint [45]. In the present study, PLOD2 and PLK1 were the two upregulated TRGs that were enriched in response to hypoxia and cell cycle pathways, respectively. Notably, qPCR analysis showed that both PLOD2 and APLK1 were significantly overexpressed in all three OSCC cell lines when compared with that in normal cells, which further validated the important role of the genes in OSCC. Therefore, we speculated that PLOD2 and APLK1 might participate in OSCC progression via responses to hypoxia and cell cycle pathways, respectively.

OSCC is the most common oral cancer, with a poor survival rate, owing to limited understanding of prognostic mechanisms. A previous study showed that age is an independent prognostic factor of overall survival in patients with OSCC [46]. Meanwhile, a comparative analysis of a large number of immune checkpoints in OSCC indicated that genes, including CD96, are significantly upregulated in patients with OSCC when compared with in healthy individuals [47]. It is believed that the overexpression of activated CD4 + T cells in patients at high risk of OSCC is a potential prognostic factor in OSCC [48]. Previous studies on the different factors affecting diseases have aimed to improve the prognosis of OSCC. Since enabling replicative immortality is a key alteration fundamental to cancer cell development, TRGs that control cell division are crucial for the occurrence and development of OSCC [49]. Several potential biomarkers for the diagnosis and treatment of human cancers, including OSCC, can be revealed [50]. CCNA2 (Cyclin A2) is a prognostic biomarker commonly overexpressed in human cancer [51]. A recent study has shown that CCNA2 is a drug-specific sensitivity-related prognostic biomarker of OSCC [52]. Li et al. indicated that downregulation of CCNA2 could suppress cell proliferation in OSCC [53]. In addition, as a member of the nuclear transporter family, KPNA2 (Karyopherin α2) often plays a key role in the nuclear cytoplasmic transport pathway of human tumor cells [54]. It has been shown that the suppression of KPNA2 can inhibit autophagy in OSCC [55]. In the present study, a prognostic model was constructed based on eight upregulated TRGs associated with OSCC: CCNB1, PDK4, PLOD2, RACGAP1, MET, PLK1, KPNA2, and CCNA2. An investigation based on the current prognostic model showed that age, immune checkpoint gene CD96, and activated CD4 + T cell infiltration were significantly different between the high- and low-risk groups, which is consistent with the findings of previous studies. Therefore, we speculate that the current prognostic model is valuable for the clinical pre-diagnosis of OSCC. TRGs, including CCNB1, PDK4, PLOD2, RACGAP1, MET, PLK1, KPNA2, and CCNA2, may serve as novel prognostic biomarkers for OSCC. Studies with large size of clinical samples are imperative in the near future.

## Conclusions

In conclusion, we constructed a novel prognostic model for OSCC based on DRGs. Moreover, PLOD2 and APLK1 may participate in OSCC progression via responses to hypoxia and cell cycle pathways, respectively. Furthermore, TRGs, including CCNB1, PDK4, PLOD2, RACGAP1, MET, PLK1, KPNA2, and CCNA2, may serve as novel prognostic biomarkers for OSCC.

## Electronic supplementary material

Below is the link to the electronic supplementary material.

Supplementary Material 1

Supplementary Material 2

Supplementary Material 3

## Data Availability

The datasets used and/or analyzed in the current study are available from the corresponding author upon reasonable request.

## List of Abbreviations

BH: Benjamini-Hochberg
DE-TRGs: Differentially expressed TRGs
DEGs: Differentially expressed genes
FC: Fold change
GEO: Gene Expression Omnibus
OSCC: Oral squamous cell carcinoma
TRF1: Telomeric Repeat Binding Factor 1
TRGs: Telomere-related genes
