# Supplementary material for: Prognostic model based on telomere-related genes predicts the risk of oral squamous cell carcinoma
Source: BMC Oral Health. 2023 Jul 14;23:484. doi: 10.1186/s12903-023-03157-x (PMC10347773; doi:10.1186/s12903-023-03157-x)
Supplement: Supplementary file 1 — Supplementary Material 1 [file 12903_2023_3157_MOESM1_ESM.docx]

Supplementary Table 1 The GO and KEGG enrichment analysis of differentially expressed telomere-related genes

| **Category** | **Term** | **Count** | **%** | **PValue** | **Genes** | **Benjamini** | **FDR** |
| --- | --- | --- | --- | --- | --- | --- | --- |
| GOTERM_BP_DIRECT | GO:0006260~DNA replication | 17 | 10.49382716 | 3.73E-15 | RMI2, FEN1, RFC4, RNASEH2A, PCNA, MCM7, DSCC1, CHTF18, RECQL4, ORC6, ORC1, CDK2, CDK1, MCM4, MCM5, MCM6, MCM2 | 4.85E-12 | 4.73E-12 |
| GOTERM_BP_DIRECT | GO:0006270~DNA replication initiation | 11 | 6.790123457 | 1.27E-14 | PRIM2, ORC6, CDC45, MCM7, ORC1, CCNE1, MCM3, MCM4, MCM5, MCM6, MCM2 | 8.27E-12 | 8.07E-12 |
| GOTERM_BP_DIRECT | GO:0006268~DNA unwinding involved in DNA replication | 9 | 5.555555556 | 7.99E-12 | RECQL4, RAD51, CDC45, MCM7, MCM3, MCM4, MCM5, MCM6, MCM2 | 3.46E-09 | 3.37E-09 |
| GOTERM_BP_DIRECT | GO:0000727~double-strand break repair via break-induced replication | 7 | 4.320987654 | 2.56E-10 | CDC45, MCM7, MCM3, MCM4, MCM5, MCM6, MCM2 | 8.30E-08 | 8.10E-08 |
| GOTERM_BP_DIRECT | GO:0030174~regulation of DNA-dependent DNA replication initiation | 7 | 4.320987654 | 8.20E-10 | MCM7, GMNN, MCM3, MCM4, MCM5, MCM6, MCM2 | 2.13E-07 | 2.08E-07 |
| GOTERM_BP_DIRECT | GO:0006267~pre-replicative complex assembly involved in nuclear cell cycle DNA replication | 6 | 3.703703704 | 1.98E-09 | MCM7, MCM3, MCM4, MCM5, MCM6, MCM2 | 4.28E-07 | 4.18E-07 |
| GOTERM_BP_DIRECT | GO:0051301~cell division | 19 | 11.72839506 | 2.88E-09 | RCC2, TUBB, CDCA8, NCAPG, HMGA2, NDC80, AURKB, CDC25B, AURKA, CCNA2, CDC20, CENPF, CCNB1, CCNE1, RAD21, CDK2, CDK1, MCM5, NEK2 | 5.34E-07 | 5.21E-07 |
| GOTERM_BP_DIRECT | GO:0000086~G2/M transition of mitotic cell cycle | 9 | 5.555555556 | 3.22E-08 | CCNA2, CCNB1, PLK1, CDK2, CDK1, PKMYT1, FOXM1, CDC25B, AURKA | 5.23E-06 | 5.10E-06 |
| GOTERM_BP_DIRECT | GO:0007049~cell cycle | 16 | 9.87654321 | 3.05E-07 | FANCI, MCM7, RCC2, USP2, HMGA2, MKI67, CHTF18, CDC25B, AURKA, KAT2B, CDC45, MCM3, MCM4, MCM5, MCM6, MCM2 | 4.41E-05 | 4.30E-05 |
| GOTERM_BP_DIRECT | GO:0000278~mitotic cell cycle | 11 | 6.790123457 | 6.14E-07 | WDHD1, CENPF, TUBB, PLK1, CDCA8, NEK2, PKMYT1, NDC80, AURKB, CDC25B, AURKA | 7.98E-05 | 7.79E-05 |
| GOTERM_BP_DIRECT | GO:0006281~DNA repair | 14 | 8.641975309 | 1.04E-06 | FANCI, WDHD1, RMI2, FEN1, RFC4, FOXM1, FANCG, RAD51AP1, RECQL4, RAD51, EXO1, CDK2, CDK1, RAD54L | 1.22E-04 | 1.19E-04 |
| GOTERM_BP_DIRECT | GO:0000281~mitotic cytokinesis | 8 | 4.938271605 | 1.32E-06 | RACGAP1, KIF4A, CFL1, PLK1, CDCA8, ECT2, MYH10, AURKB | 1.42E-04 | 1.39E-04 |
| GOTERM_BP_DIRECT | GO:0042493~response to drug | 13 | 8.024691358 | 6.00E-06 | ANXA1, MCM7, FOS, ALDH3A1, CENPF, CCNB1, RAD51, LGALS1, CDK1, DNMT3B, FOSB, RAD54L, ADA | 5.62E-04 | 5.49E-04 |
| GOTERM_BP_DIRECT | GO:0007094~mitotic spindle assembly checkpoint | 6 | 3.703703704 | 6.06E-06 | CDC20, CENPF, PLK1, TRIP13, NDC80, AURKB | 5.62E-04 | 5.49E-04 |
| GOTERM_BP_DIRECT | GO:0007052~mitotic spindle organization | 7 | 4.320987654 | 6.66E-06 | CCNB1, KIF4A, PLK1, CDCA8, NDC80, AURKB, AURKA | 5.76E-04 | 5.62E-04 |
| GOTERM_BP_DIRECT | GO:0032508~DNA duplex unwinding | 7 | 4.320987654 | 3.63E-05 | RECQL4, RFC4, ANXA1, DSCC1, MCM3, RAD54L, CHTF18 | 0.00294579 | 0.002873223 |
| GOTERM_BP_DIRECT | GO:0006271~DNA strand elongation involved in DNA replication | 4 | 2.469135802 | 4.47E-05 | RFC4, MCM7, MCM3, MCM4 | 0.003272794 | 0.003192171 |
| GOTERM_BP_DIRECT | GO:0051321~meiotic cell cycle | 7 | 4.320987654 | 4.54E-05 | RAD51AP1, RAD51, EXO1, CDK2, RAD54L, NEK2, PKMYT1 | 0.003272794 | 0.003192171 |
| GOTERM_BP_DIRECT | GO:0006468~protein phosphorylation | 15 | 9.259259259 | 7.29E-05 | PLK1, CDCA8, PIK3CD, PKMYT1, AURKB, CDC25B, AURKA, RPS6KA4, CCNB1, CCNE1, CDK2, PDK4, CDK1, NEK2, IP6K3 | 0.004981195 | 0.004858486 |
| GOTERM_BP_DIRECT | GO:0009636~response to toxic substance | 7 | 4.320987654 | 8.39E-05 | RAD51, SLC7A8, EPHX2, CDK1, DNMT3B, NEFL, FOS | 0.005370892 | 0.005238584 |
| GOTERM_BP_DIRECT | GO:0051256~mitotic spindle midzone assembly | 4 | 2.469135802 | 8.68E-05 | RACGAP1, KIF4A, CDCA8, AURKB | 0.005370892 | 0.005238584 |
| GOTERM_BP_DIRECT | GO:0001666~response to hypoxia | 9 | 5.555555556 | 1.38E-04 | EGR1, ALDH3A1, TFRC, DNMT3B, PLOD2, PLOD1, ADA, PML, HSP90B1 | 0.00816517 | 0.007964026 |
| GOTERM_BP_DIRECT | GO:1900264~positive regulation of DNA-directed DNA polymerase activity | 4 | 2.469135802 | 1.49E-04 | RFC4, PCNA, DSCC1, CHTF18 | 0.008397347 | 0.008190484 |
| GOTERM_BP_DIRECT | GO:1903801~L-leucine import into cell | 3 | 1.851851852 | 2.02E-04 | SLC7A5, SLC7A8, SLC3A2 | 0.010504314 | 0.010245547 |
| GOTERM_BP_DIRECT | GO:0098713~leucine import across plasma membrane | 3 | 1.851851852 | 2.02E-04 | SLC7A5, SLC7A8, SLC3A2 | 0.010504314 | 0.010245547 |
| GOTERM_BP_DIRECT | GO:0036297~interstrand cross-link repair | 5 | 3.086419753 | 2.65E-04 | RAD51AP1, FANCI, RAD51, FANCE, FANCG | 0.01326422 | 0.012937465 |
| GOTERM_BP_DIRECT | GO:0016572~histone phosphorylation | 4 | 2.469135802 | 2.86E-04 | RPS6KA4, CCNA2, CDK2, CDK1 | 0.013675142 | 0.013338264 |
| GOTERM_BP_DIRECT | GO:0045893~positive regulation of transcription, DNA-templated | 17 | 10.49382716 | 2.95E-04 | EGR1, AFAP1L2, FOXF2, ACTN1, ATAD2, HMGA2, FOS, FOXM1, PML, CCNA2, TBX15, KLF7, KAT2B, TF, CDK2, E2F1, TEAD2 | 0.013675142 | 0.013338264 |
| GOTERM_BP_DIRECT | GO:0006310~DNA recombination | 6 | 3.703703704 | 6.23E-04 | RECQL4, RAD51, EXO1, RAD21, RAD54L, HMGB3 | 0.027906704 | 0.027219241 |
| GOTERM_BP_DIRECT | GO:1902975~mitotic DNA replication initiation | 3 | 1.851851852 | 9.94E-04 | MCM3, MCM4, MCM2 | 0.043058978 | 0.041998249 |
| GOTERM_BP_DIRECT | GO:0000122~negative regulation of transcription from RNA polymerase II promoter | 19 | 11.72839506 | 0.001070849 | EGR1, DNMT1, PCNA, CBX3, PLK1, USP2, HMGA2, FOXM1, AURKB, TBX15, KLF7, BRMS1, CCNE1, CDK2, NFE2L3, E2F1, DNMT3B, FOSB, MAGEA4 | 0.044872015 | 0.043766623 |
| GOTERM_BP_DIRECT | GO:0010971~positive regulation of G2/M transition of mitotic cell cycle | 4 | 2.469135802 | 0.001724184 | CCNB1, RCC2, CDK1, CDC25B | 0.069991113 | 0.068266928 |
| GOTERM_BP_DIRECT | GO:1902425~positive regulation of attachment of mitotic spindle microtubules to kinetochore | 3 | 1.851851852 | 0.001836208 | KAT2B, CDCA8, AURKB | 0.070153931 | 0.068425736 |
| GOTERM_BP_DIRECT | GO:0051988~regulation of attachment of spindle microtubules to kinetochore | 3 | 1.851851852 | 0.001836208 | RACGAP1, NEK2, ECT2 | 0.070153931 | 0.068425736 |
| GOTERM_BP_DIRECT | GO:0007131~reciprocal meiotic recombination | 4 | 2.469135802 | 0.002513101 | RAD51, RAD21, RAD54L, TRIP13 | 0.093271951 | 0.090974258 |
| GOTERM_BP_DIRECT | GO:0051973~positive regulation of telomerase activity | 4 | 2.469135802 | 0.002739743 | GREM1, PARM1, NEK2, AURKB | 0.09885906 | 0.096423733 |
| GOTERM_BP_DIRECT | GO:0000070~mitotic sister chromatid segregation | 4 | 2.469135802 | 0.002978596 | PLK1, CDCA8, NEK2, NDC80 | 0.104572862 | 0.101996779 |
| GOTERM_BP_DIRECT | GO:0010389~regulation of G2/M transition of mitotic cell cycle | 3 | 1.851851852 | 0.003548598 | CENPF, CDK2, AURKA | 0.12130601 | 0.118317717 |
| GOTERM_BP_DIRECT | GO:0045892~negative regulation of transcription, DNA-templated | 13 | 8.024691358 | 0.0036783 | FOXF2, CBX3, GMNN, HMGA2, FOXM1, PML, GREM1, CENPF, BRMS1, BASP1, MYF6, E2F1, PITX1 | 0.122515691 | 0.119497599 |
| GOTERM_BP_DIRECT | GO:0090267~positive regulation of mitotic cell cycle spindle assembly checkpoint | 3 | 1.851851852 | 0.004235304 | CDCA8, NDC80, AURKB | 0.137541482 | 0.134153239 |
| GOTERM_BP_DIRECT | GO:0032467~positive regulation of cytokinesis | 4 | 2.469135802 | 0.00467932 | RACGAP1, ECT2, AURKB, CDC25B | 0.148254551 | 0.144602399 |
| GOTERM_BP_DIRECT | GO:0006275~regulation of DNA replication | 4 | 2.469135802 | 0.006865993 | CCNA2, PCNA, DSCC1, GMNN | 0.212355356 | 0.207124123 |
| GOTERM_BP_DIRECT | GO:0000724~double-strand break repair via homologous recombination | 5 | 3.086419753 | 0.007679203 | RECQL4, RAD51AP1, RMI2, FEN1, RAD51 | 0.231983368 | 0.226268612 |
| GOTERM_BP_DIRECT | GO:0000079~regulation of cyclin-dependent protein serine/threonine kinase activity | 4 | 2.469135802 | 0.009569363 | CCNA2, CCNB1, CCNE1, PKMYT1 | 0.282513696 | 0.275554159 |
| GOTERM_BP_DIRECT | GO:0045944~positive regulation of transcription from RNA polymerase II promoter | 19 | 11.72839506 | 0.010363684 | TOP2A, EGR1, FOXF2, ATAD2, HMGA2, FOS, FOXM1, KLF15, RGMA, GREM1, RPS6KA4, KLF7, KAT2B, MYF6, E2F1, FOSB, MET, PITX1, TEAD2 | 0.299165017 | 0.291795286 |
| GOTERM_BP_DIRECT | GO:0048146~positive regulation of fibroblast proliferation | 4 | 2.469135802 | 0.011670227 | CCNA2, CCNB1, E2F1, PML | 0.30955329 | 0.301927651 |
| GOTERM_BP_DIRECT | GO:0007064~mitotic sister chromatid cohesion | 3 | 1.851851852 | 0.01167676 | CDC20, DSCC1, RAD21 | 0.30955329 | 0.301927651 |
| GOTERM_BP_DIRECT | GO:0051412~response to corticosterone | 3 | 1.851851852 | 0.01167676 | FOSB, NEFL, FOS | 0.30955329 | 0.301927651 |
| GOTERM_BP_DIRECT | GO:0044849~estrous cycle | 3 | 1.851851852 | 0.01167676 | EGR1, PCNA, ANXA1 | 0.30955329 | 0.301927651 |
| GOTERM_BP_DIRECT | GO:0043066~negative regulation of apoptotic process | 11 | 6.790123457 | 0.012144765 | GREM1, ANXA1, TFRC, CFL1, PLK1, ANXA5, CDK1, HMGA2, HSP90B1, AURKA, MAGEA4 | 0.315521008 | 0.307748358 |
| GOTERM_BP_DIRECT | GO:0006351~transcription, DNA-templated | 5 | 3.086419753 | 0.012744967 | CCNA2, DNMT1, PPP1R1B, CDK2, E2F1 | 0.324621808 | 0.316624966 |
| GOTERM_BP_DIRECT | GO:0009615~response to virus | 5 | 3.086419753 | 0.013548798 | IFITM1, CFL1, HMGA2, ISG15, CCT5 | 0.338459405 | 0.330121683 |
| GOTERM_BP_DIRECT | GO:0009410~response to xenobiotic stimulus | 7 | 4.320987654 | 0.015103263 | CENPF, RAD51, CDK1, FOSB, RAD54L, FOS, ENO2 | 0.353718794 | 0.345005167 |
| GOTERM_BP_DIRECT | GO:0007346~regulation of mitotic cell cycle | 4 | 2.469135802 | 0.015281915 | CDC20, AFAP1L2, PLK1, GMNN | 0.353718794 | 0.345005167 |
| GOTERM_BP_DIRECT | GO:0001556~oocyte maturation | 3 | 1.851851852 | 0.015299442 | CCNB1, TRIP13, CDC25B | 0.353718794 | 0.345005167 |
| GOTERM_BP_DIRECT | GO:0007088~regulation of mitotic nuclear division | 3 | 1.851851852 | 0.015299442 | NEK2, PKMYT1, MKI67 | 0.353718794 | 0.345005167 |
| GOTERM_BP_DIRECT | GO:0015829~valine transport | 2 | 1.234567901 | 0.016446578 | SLC7A5, SLC7A8 | 0.353718794 | 0.345005167 |
| GOTERM_BP_DIRECT | GO:0015823~phenylalanine transport | 2 | 1.234567901 | 0.016446578 | SLC7A5, SLC3A2 | 0.353718794 | 0.345005167 |
| GOTERM_BP_DIRECT | GO:1905784~regulation of anaphase-promoting complex-dependent catabolic process | 2 | 1.234567901 | 0.016446578 | PLK1, CDK2 | 0.353718794 | 0.345005167 |
| GOTERM_BP_DIRECT | GO:0044772~mitotic cell cycle phase transition | 3 | 1.851851852 | 0.016600814 | CCNA2, CCNB1, CCNE1 | 0.353718794 | 0.345005167 |
| GOTERM_BP_DIRECT | GO:0071466~cellular response to xenobiotic stimulus | 4 | 2.469135802 | 0.016610351 | PCNA, MCM7, TFRC, E2F1 | 0.353718794 | 0.345005167 |
| GOTERM_BP_DIRECT | GO:0046686~response to cadmium ion | 3 | 1.851851852 | 0.022253012 | DTYMK, PCNA, CDK1 | 0.449504968 | 0.438431712 |
| GOTERM_BP_DIRECT | GO:0031507~heterochromatin assembly | 3 | 1.851851852 | 0.022253012 | CBX3, HMGA2, LMNB1 | 0.449504968 | 0.438431712 |
| GOTERM_BP_DIRECT | GO:0071398~cellular response to fatty acid | 3 | 1.851851852 | 0.022253012 | CCNB1, E2F1, PDK4 | 0.449504968 | 0.438431712 |
| GOTERM_BP_DIRECT | GO:0007568~aging | 6 | 3.703703704 | 0.02257076 | ALDH3A1, TFRC, FOS, CTSC, ADA, AURKB | 0.449504968 | 0.438431712 |
| GOTERM_BP_DIRECT | GO:0070301~cellular response to hydrogen peroxide | 4 | 2.469135802 | 0.023367754 | PCNA, ANXA1, CDK1, ECT2 | 0.449504968 | 0.438431712 |
| GOTERM_BP_DIRECT | GO:0010033~response to organic substance | 3 | 1.851851852 | 0.023773666 | ANXA5, AMPD1, CTSC | 0.449504968 | 0.438431712 |
| GOTERM_BP_DIRECT | GO:0003009~skeletal muscle contraction | 3 | 1.851851852 | 0.023773666 | JSRP1, TNNC1, TNNC2 | 0.449504968 | 0.438431712 |
| GOTERM_BP_DIRECT | GO:0043988~histone H3-S28 phosphorylation | 2 | 1.234567901 | 0.024568786 | RPS6KA4, AURKB | 0.449504968 | 0.438431712 |
| GOTERM_BP_DIRECT | GO:0051987~positive regulation of attachment of spindle microtubules to kinetochore | 2 | 1.234567901 | 0.024568786 | CCNB1, RCC2 | 0.449504968 | 0.438431712 |
| GOTERM_BP_DIRECT | GO:1904273~L-alanine import across plasma membrane | 2 | 1.234567901 | 0.024568786 | SLC7A8, SLC3A2 | 0.449504968 | 0.438431712 |
| GOTERM_BP_DIRECT | GO:0007059~chromosome segregation | 4 | 2.469135802 | 0.025043183 | TOP2A, CENPF, NEK2, NDC80 | 0.450837843 | 0.439731753 |
| GOTERM_BP_DIRECT | GO:0006306~DNA methylation | 3 | 1.851851852 | 0.025335768 | DNMT1, DNMT3B, FOS | 0.450837843 | 0.439731753 |
| GOTERM_BP_DIRECT | GO:0048511~rhythmic process | 4 | 2.469135802 | 0.025904588 | TOP2A, KAT2B, CBX3, CDK1 | 0.454730545 | 0.443528561 |
| GOTERM_BP_DIRECT | GO:0010628~positive regulation of gene expression | 10 | 6.172839506 | 0.026822824 | EGR1, DNMT1, TFRC, EPHX2, CDK1, E2F1, DNMT3B, HMGA2, PIK3CD, MSN | 0.46043636 | 0.449093817 |
| GOTERM_BP_DIRECT | GO:0043278~response to morphine | 3 | 1.851851852 | 0.02693854 | PPP1R1B, FOSB, ADA | 0.46043636 | 0.449093817 |
| GOTERM_BP_DIRECT | GO:0009887~animal organ morphogenesis | 5 | 3.086419753 | 0.027628406 | GAMT, GREM1, FOXF2, FHL1, GMNN | 0.464087436 | 0.452654951 |
| GOTERM_BP_DIRECT | GO:0045931~positive regulation of mitotic cell cycle | 3 | 1.851851852 | 0.028581212 | CCNB1, USP2, CDC25B | 0.464087436 | 0.452654951 |
| GOTERM_BP_DIRECT | GO:0043392~negative regulation of DNA binding | 3 | 1.851851852 | 0.028581212 | E2F1, HMGA2, NEK2 | 0.464087436 | 0.452654951 |
| GOTERM_BP_DIRECT | GO:0006298~mismatch repair | 3 | 1.851851852 | 0.028581212 | RNASEH2A, PCNA, EXO1 | 0.464087436 | 0.452654951 |
| GOTERM_BP_DIRECT | GO:1900182~positive regulation of protein localization to nucleus | 3 | 1.851851852 | 0.030263026 | TFRC, PLK1, CDK1 | 0.47616872 | 0.464438621 |
| GOTERM_BP_DIRECT | GO:0032212~positive regulation of telomere maintenance via telomerase | 3 | 1.851851852 | 0.031983231 | NEK2, CCT5, AURKB | 0.47616872 | 0.464438621 |
| GOTERM_BP_DIRECT | GO:0007057~spindle assembly involved in female meiosis I | 2 | 1.234567901 | 0.032624339 | NDC80, AURKA | 0.47616872 | 0.464438621 |
| GOTERM_BP_DIRECT | GO:0043137~DNA replication, removal of RNA primer | 2 | 1.234567901 | 0.032624339 | FEN1, RNASEH2A | 0.47616872 | 0.464438621 |
| GOTERM_BP_DIRECT | GO:0015827~tryptophan transport | 2 | 1.234567901 | 0.032624339 | SLC7A8, SLC3A2 | 0.47616872 | 0.464438621 |
| GOTERM_BP_DIRECT | GO:0045876~positive regulation of sister chromatid cohesion | 2 | 1.234567901 | 0.032624339 | FEN1, RAD21 | 0.47616872 | 0.464438621 |
| GOTERM_BP_DIRECT | GO:1901970~positive regulation of mitotic sister chromatid separation | 2 | 1.234567901 | 0.032624339 | CDCA8, AURKB | 0.47616872 | 0.464438621 |
| GOTERM_BP_DIRECT | GO:1905448~positive regulation of mitochondrial ATP synthesis coupled electron transport | 2 | 1.234567901 | 0.032624339 | CCNB1, CDK1 | 0.47616872 | 0.464438621 |
| GOTERM_BP_DIRECT | GO:0090402~oncogene-induced cell senescence | 2 | 1.234567901 | 0.032624339 | HMGA2, PML | 0.47616872 | 0.464438621 |
| GOTERM_BP_DIRECT | GO:0007095~mitotic G2 DNA damage checkpoint | 3 | 1.851851852 | 0.035535865 | PLK1, CDK1, HMGA2 | 0.512900992 | 0.500266018 |
| GOTERM_BP_DIRECT | GO:0065003~macromolecular complex assembly | 5 | 3.086419753 | 0.037439778 | CCNB1, GMNN, CDK1, PML, TEAD2 | 0.534442546 | 0.52127691 |
| GOTERM_BP_DIRECT | GO:0007265~Ras protein signal transduction | 4 | 2.469135802 | 0.038529453 | CCNA2, DNMT1, CDK2, RRAS2 | 0.543889699 | 0.530491338 |
| GOTERM_BP_DIRECT | GO:1903490~positive regulation of mitotic cytokinesis | 2 | 1.234567901 | 0.04061378 | CDCA8, AURKB | 0.543889699 | 0.530491338 |
| GOTERM_BP_DIRECT | GO:0090116~C-5 methylation of cytosine | 2 | 1.234567901 | 0.04061378 | DNMT1, DNMT3B | 0.543889699 | 0.530491338 |
| GOTERM_BP_DIRECT | GO:0032263~GMP salvage | 2 | 1.234567901 | 0.04061378 | AMPD1, ADA | 0.543889699 | 0.530491338 |
| GOTERM_BP_DIRECT | GO:0015820~leucine transport | 2 | 1.234567901 | 0.04061378 | SLC7A8, SLC3A2 | 0.543889699 | 0.530491338 |
| GOTERM_BP_DIRECT | GO:0033031~positive regulation of neutrophil apoptotic process | 2 | 1.234567901 | 0.04061378 | ANXA1, PIK3CD | 0.543889699 | 0.530491338 |
| GOTERM_BP_DIRECT | GO:0030855~epithelial cell differentiation | 4 | 2.469135802 | 0.042939569 | PCNA, CDK1, TAGLN2, KRT10 | 0.569168368 | 0.555147285 |
| GOTERM_BP_DIRECT | GO:0071479~cellular response to ionizing radiation | 3 | 1.851851852 | 0.045038647 | RAD51AP1, RAD51, ECT2 | 0.579259428 | 0.564989757 |
| GOTERM_BP_DIRECT | GO:1901796~regulation of signal transduction by p53 class mediator | 3 | 1.851851852 | 0.045038647 | USP2, AURKB, AURKA | 0.579259428 | 0.564989757 |
| GOTERM_BP_DIRECT | GO:0030316~osteoclast differentiation | 3 | 1.851851852 | 0.045038647 | TF, TFRC, FOS | 0.579259428 | 0.564989757 |
| GOTERM_BP_DIRECT | GO:0032465~regulation of cytokinesis | 3 | 1.851851852 | 0.047040125 | PLK1, AURKB, AURKA | 0.593253614 | 0.578639207 |
| GOTERM_BP_DIRECT | GO:0097421~liver regeneration | 3 | 1.851851852 | 0.047040125 | SLC7A5, PCNA, AURKA | 0.593253614 | 0.578639207 |
| GOTERM_BP_DIRECT | GO:0051573~negative regulation of histone H3-K9 methylation | 2 | 1.234567901 | 0.04853765 | DNMT1, DNMT3B | 0.606253914 | 0.591319253 |
| GOTERM_CC_DIRECT | GO:0005654~nucleoplasm | 81 | 50 | 1.30E-18 | TOP2A, FEN1, MCM7, DSCC1, GMNN, FOXM1, CHTF18, MKI67, LMNB1, RPS6KA4, CDC20, EXO1, DNMT3B, NEK2, KPNA2, TEAD2, CTSC, WDHD1, RMI2, ANXA1, ACOT7, RFC4, USP2, FOS, KLF15, CDC25B, CCNA2, KAT2B, ACLY, DDX39A, BRMS1, CCNE1, RRAGD, MCM3, MCM4, MCM5, MCM6, ASF1B, MCM2, SNRPB, PRIM2, DNMT1, RNASEH2A, PCNA, PARM1, CDCA8, SLC3A2, PKMYT1, AURKB, AURKA, RAD51AP1, RECQL4, CCNB1, ORC6, SOCS1, CDC45, ORC1, RACGAP1, RAD21, E2F1, RAD54L, ECT2, FANCI, EGR1, CBX3, PLK1, ATAD2, HMGA2, ISG15, FANCE, FANCG, NDC80, PML, KLF7, CENPF, RAD51, KIF4A, CDK2, MYF6, CDK1, FOSB | 4.20E-16 | 3.76E-16 |
| GOTERM_CC_DIRECT | GO:0005634~nucleus | 95 | 58.64197531 | 3.51E-15 | TOP2A, FEN1, CLIC3, MCM7, TFRC, CALML6, DSCC1, FHL1, GMNN, HMGB3, FOXM1, CHTF18, MKI67, LMNB1, RPS6KA4, LGALS1, BASP1, EXO1, CFL1, COTL1, DNMT3B, NEK2, KPNA2, PITX1, TEAD2, MAGEA4, GAMT, WDHD1, RMI2, ANXA1, RFC4, TUBB, ACTN1, FOS, KLF15, CDC25B, CCNA2, KAT2B, AZGP1, DDX39A, BRMS1, CCNE1, RRAGD, MCM3, MCM4, MCM5, MCM6, ASF1B, MCM2, SNRPB, DTYMK, DNMT1, PCNA, NCAPG, PKMYT1, USP18, AURKB, AURKA, HSP90B1, RAD51AP1, RECQL4, CCNB1, CDC45, ORC1, RACGAP1, RAD21, E2F1, RAD54L, IGF2BP2, ECT2, MYH10, IP6K3, EGR1, FOXF2, CBX3, RCC2, PLK1, ATAD2, HMGA2, MSN, ISG15, KRT10, FANCE, NDC80, PML, KLF7, CENPF, RAD51, PPP1R1B, CDK2, NFE2L3, MYF6, CDK1, FOSB, TRIP13 | 5.67E-13 | 5.08E-13 |
| GOTERM_CC_DIRECT | GO:0005829~cytosol | 88 | 54.32098765 | 1.15E-13 | MCM7, FHL1, GMNN, PIK3CD, CHTF18, ENO2, LMNB1, RPS6KA4, CDC20, LGALS1, CFL1, COTL1, NEFL, NEK2, KPNA2, TEAD2, GAMT, RMI2, ANXA1, ACOT7, TUBB, ACTN1, TNNC1, SIGMAR1, ANXA5, TNNC2, AMPD1, FOS, CDC25B, ENAH, RAB32, CCNA2, KAT2B, ACLY, SLC7A5, ALDH3A1, CCNE1, RRAGD, ALDH1A1, RAB38, MCM5, TAGLN2, IDO1, SNRPB, DTYMK, RNASEH2A, PARM1, EIF5A2, NCAPG, CDCA8, HMMR, PKMYT1, USP18, AURKB, AURKA, HSP90B1, CCNB1, ORC6, SOCS1, ORC1, RACGAP1, GMDS, CALD1, RAD21, IGF2BP2, ECT2, MYH10, IP6K3, CCT5, FANCI, AFAP1L2, MYO10, EPHX2, RCC2, PLK1, MSN, ISG15, KRT10, FANCG, NDC80, PML, KLF7, CENPF, KIF4A, PPP1R1B, CDK2, CDK1, ADA | 1.24E-11 | 1.11E-11 |
| GOTERM_CC_DIRECT | GO:0000781~chromosome, telomeric region | 16 | 9.87654321 | 6.72E-12 | FEN1, PCNA, MCM7, CBX3, PML, RAD51AP1, RECQL4, RAD51, ORC1, CDK2, MCM3, CDK1, MCM4, MCM5, MCM6, MCM2 | 5.43E-10 | 4.86E-10 |
| GOTERM_CC_DIRECT | GO:0071162~CMG complex | 7 | 4.320987654 | 9.28E-11 | CDC45, MCM7, MCM3, MCM4, MCM5, MCM6, MCM2 | 5.99E-09 | 5.36E-09 |
| GOTERM_CC_DIRECT | GO:0030496~midbody | 14 | 8.641975309 | 2.42E-09 | RCC2, PLK1, CDCA8, AURKB, AURKA, HSP90B1, CENPF, RACGAP1, KIF4A, RAD21, CDK1, NEK2, ECT2, MYH10 | 1.30E-07 | 1.17E-07 |
| GOTERM_CC_DIRECT | GO:0000785~chromatin | 30 | 18.51851852 | 3.04E-09 | PCNA, MCM7, DSCC1, FOXM1, RAD51AP1, BASP1, RAD21, E2F1, PITX1, TEAD2, FANCI, EGR1, FOXF2, CBX3, PLK1, HMGA2, FOS, FANCE, KLF15, FANCG, PML, TBX15, KLF7, CENPF, RAD51, NFE2L3, MYF6, FOSB, MCM2, ASF1B | 1.36E-07 | 1.22E-07 |
| GOTERM_CC_DIRECT | GO:0042555~MCM complex | 6 | 3.703703704 | 3.36E-09 | MCM7, MCM3, MCM4, MCM5, MCM6, MCM2 | 1.36E-07 | 1.22E-07 |
| GOTERM_CC_DIRECT | GO:0005813~centrosome | 21 | 12.96296296 | 1.09E-08 | PCNA, PLK1, USP2, HMMR, NDC80, CDC25B, AURKA, CDC20, KAT2B, CENPF, CCNB1, CDC45, CCNE1, RRAGD, CDK2, MCM3, E2F1, CDK1, NEK2, CCT5, CTSC | 3.91E-07 | 3.50E-07 |
| GOTERM_CC_DIRECT | GO:0016020~membrane | 44 | 27.16049383 | 5.91E-07 | IFITM1, FEN1, TENM3, CLIC3, MCM7, TFRC, NCAPG, PIK3CD, SLC3A2, HMMR, PKMYT1, MKI67, ENO2, CHTF18, HSP90B1, LMNB1, RECQL4, CCNB1, ORC6, RAD21, CFL1, KPNA2, CTSC, FANCI, RCC2, USP2, SIGMAR1, ANXA5, RRAS2, KRT10, FOS, NDC80, RAB32, SLC7A5, ACLY, DDX39A, KIF4A, MCM3, CDK1, MCM4, RAB38, MCM5, MET, ADA | 1.76E-05 | 1.57E-05 |
| GOTERM_CC_DIRECT | GO:0000775~chromosome, centromeric region | 8 | 4.938271605 | 5.98E-07 | TOP2A, CENPF, CBX3, DSCC1, RAD21, CDCA8, NDC80, AURKB | 1.76E-05 | 1.57E-05 |
| GOTERM_CC_DIRECT | GO:0005694~chromosome | 13 | 8.024691358 | 8.48E-07 | MCM7, HMGB3, MKI67, RAD51AP1, RECQL4, KIF4A, RAD21, MCM3, MCM4, MCM5, TRIP13, MCM6, MCM2 | 2.28E-05 | 2.04E-05 |
| GOTERM_CC_DIRECT | GO:0005737~cytoplasm | 73 | 45.0617284 | 9.76E-07 | TOP2A, IL1RN, CLIC3, CALML6, FHL1, GMNN, PIK3CD, HMGB3, PCSK9, RPS6KA4, LASP1, LGALS1, BASP1, CFL1, NEFL, NEK2, KPNA2, PITX1, TMSB10, GAMT, ARRDC4, WDHD1, ANXA1, ARL14, TUBB, ACTN1, USP2, ANXA5, CDC25B, ENAH, CCNA2, ACLY, ALDH3A1, DDX39A, BRMS1, SLC7A8, CCNE1, RRAGD, ALDH1A1, MCM3, IDO1, MCM2, SNRPB, DTYMK, NCAPG, HMMR, RECQL4, CCNB1, SOCS1, CDC45, GMDS, E2F1, IGF2BP2, ECT2, MYH10, IP6K3, CCT5, FANCI, EGR1, AFAP1L2, PLK1, MSN, ISG15, KRT10, PML, CENPF, RAD51, KIF4A, PPP1R1B, CDK2, CDK1, CCM2, NAGS | 2.42E-05 | 2.17E-05 |
| GOTERM_CC_DIRECT | GO:0000307~cyclin-dependent protein kinase holoenzyme complex | 6 | 3.703703704 | 1.44E-05 | CCNA2, CCNB1, PCNA, CCNE1, CDK2, CDK1 | 3.30E-04 | 2.95E-04 |
| GOTERM_CC_DIRECT | GO:0070062~extracellular exosome | 37 | 22.83950617 | 1.53E-05 | IL1RN, PCNA, CLIC3, TFRC, PLOD2, SLC3A2, PLOD1, ENO2, HSP90B1, LGALS1, RACGAP1, GMDS, ALDH2, BASP1, CFL1, COTL1, MYH10, CCT5, CTSC, ACOT7, ANXA1, EPHX2, ACTN1, TUBB, ATAD2, ANXA5, RRAS2, MSN, KRT10, SLC7A5, ACLY, TF, AZGP1, ALDH1A1, CDK1, TAGLN2, DSG2 | 3.30E-04 | 2.95E-04 |
| GOTERM_CC_DIRECT | GO:0051233~spindle midzone | 5 | 3.086419753 | 4.00E-05 | RACGAP1, PLK1, CDCA8, AURKB, AURKA | 8.07E-04 | 7.22E-04 |
| GOTERM_CC_DIRECT | GO:0000793~condensed chromosome | 5 | 3.086419753 | 5.48E-05 | TOP2A, RAD51, CDK2, NCAPG, MKI67 | 0.001041469 | 9.32E-04 |
| GOTERM_CC_DIRECT | GO:0000940~condensed chromosome outer kinetochore | 4 | 2.469135802 | 9.79E-05 | CENPF, CCNB1, PLK1, NDC80 | 0.00175627 | 0.0015714 |
| GOTERM_CC_DIRECT | GO:0005819~spindle | 8 | 4.938271605 | 1.66E-04 | CDC20, CENPF, RACGAP1, CBX3, PLK1, HMMR, AURKB, AURKA | 0.002823023 | 0.002525862 |
| GOTERM_CC_DIRECT | GO:0005876~spindle microtubule | 5 | 3.086419753 | 3.82E-04 | KIF4A, PLK1, CDK1, AURKB, AURKA | 0.006175711 | 0.005525636 |
| GOTERM_CC_DIRECT | GO:0032991~macromolecular complex | 16 | 9.87654321 | 4.20E-04 | TOP2A, IFITM1, FEN1, ANXA1, TUBB, CDCA8, HSP90B1, RAD51AP1, CDC20, KAT2B, RAD51, E2F1, RAD54L, NEK2, CCM2, ASF1B | 0.006464416 | 0.005783951 |
| GOTERM_CC_DIRECT | GO:0009986~cell surface | 15 | 9.259259259 | 4.98E-04 | ANXA1, TFRC, PCSK9, MSN, SLC3A2, KRT10, HMMR, ENO2, RGMA, GREM1, TF, LGALS1, DSG2, MET, ADA | 0.007199934 | 0.006442046 |
| GOTERM_CC_DIRECT | GO:0000922~spindle pole | 7 | 4.320987654 | 5.35E-04 | CDC20, CENPF, CCNB1, RAD21, PLK1, NEK2, CDC25B | 0.007199934 | 0.006442046 |
| GOTERM_CC_DIRECT | GO:0016363~nuclear matrix | 7 | 4.320987654 | 5.35E-04 | CENPF, BASP1, KIF4A, RAD21, CFL1, PML, LMNB1 | 0.007199934 | 0.006442046 |
| GOTERM_CC_DIRECT | GO:0009925~basal plasma membrane | 5 | 3.086419753 | 6.75E-04 | SLC7A5, TF, SLC7A8, SLC3A2, MET | 0.008717384 | 0.007799765 |
| GOTERM_CC_DIRECT | GO:0001673~male germ cell nucleus | 5 | 3.086419753 | 7.27E-04 | TOP2A, RAD51, PCNA, CDK2, TRIP13 | 0.009025824 | 0.008075737 |
| GOTERM_CC_DIRECT | GO:0032133~chromosome passenger complex | 3 | 1.851851852 | 8.92E-04 | CDCA8, AURKB, AURKA | 0.010670724 | 0.00954749 |
| GOTERM_CC_DIRECT | GO:0031390~Ctf18 RFC-like complex | 3 | 1.851851852 | 0.001647985 | RFC4, DSCC1, CHTF18 | 0.019010681 | 0.017009556 |
| GOTERM_CC_DIRECT | GO:0031232~extrinsic component of external side of plasma membrane | 3 | 1.851851852 | 0.002107975 | TF, ANXA1, PCSK9 | 0.022695859 | 0.020306821 |
| GOTERM_CC_DIRECT | GO:0005664~nuclear origin of replication recognition complex | 3 | 1.851851852 | 0.002107975 | ORC6, ORC1, MCM2 | 0.022695859 | 0.020306821 |
| GOTERM_CC_DIRECT | GO:0031982~vesicle | 7 | 4.320987654 | 0.002494318 | TF, ANXA1, BASP1, CFL1, MSN, RAB38, TAGLN2 | 0.025989183 | 0.02325348 |
| GOTERM_CC_DIRECT | GO:0000794~condensed nuclear chromosome | 4 | 2.469135802 | 0.002995622 | RAD51, RAD21, NCAPG, NEK2 | 0.030237064 | 0.027054215 |
| GOTERM_CC_DIRECT | GO:0072686~mitotic spindle | 6 | 3.703703704 | 0.004901309 | KAT2B, RACGAP1, TUBB, CDK1, ECT2, AURKA | 0.047973417 | 0.042923584 |
| GOTERM_CC_DIRECT | GO:0005925~focal adhesion | 10 | 6.172839506 | 0.00624146 | ENAH, LASP1, ANXA1, ACTN1, CFL1, FHL1, ANXA5, RRAS2, MSN, HSP90B1 | 0.059293871 | 0.05305241 |
| GOTERM_CC_DIRECT | GO:0000779~condensed chromosome, centromeric region | 3 | 1.851851852 | 0.006778863 | CBX3, NCAPG, AURKB | 0.062559217 | 0.055974037 |
| GOTERM_CC_DIRECT | GO:0000776~kinetochore | 6 | 3.703703704 | 0.007887288 | KAT2B, CENPF, PLK1, NEK2, NDC80, AURKB | 0.070766502 | 0.063317397 |
| GOTERM_CC_DIRECT | GO:0000228~nuclear chromosome | 4 | 2.469135802 | 0.008682343 | TOP2A, RAD51, E2F1, HMGA2 | 0.07421843 | 0.066405963 |
| GOTERM_CC_DIRECT | GO:0042470~melanosome | 5 | 3.086419753 | 0.00873158 | RAB32, TFRC, RAB38, SLC3A2, HSP90B1 | 0.07421843 | 0.066405963 |
| GOTERM_CC_DIRECT | GO:0005769~early endosome | 8 | 4.938271605 | 0.010786991 | RAB32, ARRDC4, TF, PARM1, ANXA1, TFRC, PCSK9, RAB38 | 0.089338413 | 0.07993437 |
| GOTERM_CC_DIRECT | GO:0090575~RNA polymerase II transcription factor complex | 5 | 3.086419753 | 0.015070824 | CBX3, NFE2L3, MYF6, E2F1, FOS | 0.119743805 | 0.107139194 |
| GOTERM_CC_DIRECT | GO:1990184~amino acid transport complex | 2 | 1.234567901 | 0.015570402 | SLC7A5, SLC3A2 | 0.119743805 | 0.107139194 |
| GOTERM_CC_DIRECT | GO:0097125~cyclin B1-CDK1 complex | 2 | 1.234567901 | 0.015570402 | CCNB1, CDK1 | 0.119743805 | 0.107139194 |
| GOTERM_CC_DIRECT | GO:0015630~microtubule cytoskeleton | 6 | 3.703703704 | 0.019349027 | TUBB, PLK1, CDCA8, HMMR, AURKB, AURKA | 0.145342692 | 0.130043462 |
| GOTERM_CC_DIRECT | GO:0031528~microvillus membrane | 3 | 1.851851852 | 0.021465218 | SLC7A5, SLC7A8, MSN | 0.157574216 | 0.140987457 |
| GOTERM_CC_DIRECT | GO:0097149~centralspindlin complex | 2 | 1.234567901 | 0.023265015 | RACGAP1, ECT2 | 0.163360868 | 0.146164987 |
| GOTERM_CC_DIRECT | GO:0097134~cyclin E1-CDK2 complex | 2 | 1.234567901 | 0.023265015 | CCNE1, CDK2 | 0.163360868 | 0.146164987 |
| GOTERM_CC_DIRECT | GO:0005856~cytoskeleton | 10 | 6.172839506 | 0.026593527 | ENAH, CALD1, BASP1, KIF4A, TUBB, MSN, TAGLN2, KRT10, IGF2BP2, TMSB10 | 0.182759769 | 0.163521898 |
| GOTERM_CC_DIRECT | GO:0030426~growth cone | 5 | 3.086419753 | 0.029636531 | BASP1, CFL1, SIGMAR1, NEFL, ENO2 | 0.199429157 | 0.178436614 |
| GOTERM_CC_DIRECT | GO:0097431~mitotic spindle pole | 3 | 1.851851852 | 0.035513157 | PLK1, AURKB, AURKA | 0.234096933 | 0.209455151 |
| GOTERM_CC_DIRECT | GO:0048471~perinuclear region of cytoplasm | 12 | 7.407407407 | 0.03745789 | CDC20, HHATL, CENPF, TF, RAD51, TFRC, USP2, MCM3, PCSK9, MSN, HSP90B1, AURKA | 0.234482104 | 0.209799777 |
| GOTERM_CC_DIRECT | GO:0097124~cyclin A2-CDK2 complex | 2 | 1.234567901 | 0.038475392 | CCNA2, CDK2 | 0.234482104 | 0.209799777 |
| GOTERM_CC_DIRECT | GO:0045120~pronucleus | 2 | 1.234567901 | 0.038475392 | CENPF, AURKA | 0.234482104 | 0.209799777 |
| GOTERM_CC_DIRECT | GO:0005658~alpha DNA polymerase:primase complex | 2 | 1.234567901 | 0.038475392 | PRIM2, MCM3 | 0.234482104 | 0.209799777 |
| GOTERM_CC_DIRECT | GO:0016323~basolateral plasma membrane | 6 | 3.703703704 | 0.039397916 | SLC7A5, ANXA1, SLC7A8, TFRC, MSN, SLC3A2 | 0.235657907 | 0.210851811 |
| GOTERM_CC_DIRECT | GO:0005874~microtubule | 7 | 4.320987654 | 0.043256229 | RACGAP1, RCC2, KIF4A, TUBB, NEK2, CCT5, AURKA | 0.254032038 | 0.227291823 |
| GOTERM_CC_DIRECT | GO:0000808~origin recognition complex | 2 | 1.234567901 | 0.045992074 | ORC6, ORC1 | 0.265275713 | 0.237351953 |
| GOTERM_MF_DIRECT | GO:0005515~protein binding | 148 | 91.35802469 | 3.50E-14 | IFITM1, IL1RN, TFRC, CALML6, DSCC1, GMNN, PLOD1, MKI67, ENO2, RPS6KA4, CDC20, LGALS1, BASP1, CFL1, PHYHD1, PDK4, COTL1, NEK2, KPNA2, PITX1, TMSB10, MAGEA4, GAMT, ARRDC4, WDHD1, ACOT7, ARL14, ACTN1, USP2, CDC25B, RAB32, ACLY, ALDH3A1, AZGP1, DDX39A, BRMS1, CCNE1, RRAGD, RAB38, TAGLN2, ASF1B, SNRPB, DNMT1, PARM1, EIF5A2, CDCA8, NCAPG, HMMR, PKMYT1, HSP90B1, RAD51AP1, CCNB1, ORC6, SOCS1, ORC1, RACGAP1, CALD1, RAD21, SERPINH1, ECT2, MYH10, IP6K3, FANCI, HHATL, EGR1, CBX3, RCC2, PLK1, MSN, HMGA2, ISG15, FANCG, NDC80, TF, KIF4A, PPP1R1B, CDK2, NFE2L3, CDK1, ADA, TOP2A, FEN1, CLIC3, MCM7, FHL1, HMGB3, PIK3CD, PCSK9, FOXM1, CHTF18, MYOM2, LMNB1, LASP1, EXO1, DNMT3B, NEFL, TEAD2, CTSC, RMI2, ANXA1, RFC4, TUBB, TNNC1, SIGMAR1, ANXA5, TNNC2, AMPD1, RRAS2, FOS, KLF15, RGMA, CCNA2, ENAH, GREM1, KAT2B, SLC7A5, SLC7A8, ALDH1A1, MCM3, MCM4, MCM5, MCM6, MET, MCM2, PRIM2, PCNA, SLC3A2, USP18, AURKB, AURKA, RECQL4, CDC45, GMDS, E2F1, RAD54L, IGF2BP2, CCT5, MYO10, ATAD2, KRT10, PML, TBX15, CENPF, RAD51, JSRP1, FOSB, CCM2, TRIP13 | 9.87E-12 | 9.37E-12 |
| GOTERM_MF_DIRECT | GO:0017116~single-stranded DNA-dependent ATP-dependent DNA helicase activity | 10 | 6.172839506 | 5.00E-14 | RAD51, RFC4, MCM7, DSCC1, MCM3, MCM4, MCM5, MCM6, CHTF18, MCM2 | 9.87E-12 | 9.37E-12 |
| GOTERM_MF_DIRECT | GO:0003677~DNA binding | 40 | 24.69135802 | 1.82E-12 | TOP2A, PRIM2, DNMT1, FEN1, PCNA, MCM7, DSCC1, HMGB3, MKI67, FOXM1, CHTF18, RAD51AP1, ORC6, ORC1, EXO1, RAD21, E2F1, DNMT3B, RAD54L, PITX1, TEAD2, FANCI, WDHD1, EGR1, RMI2, RFC4, FOXF2, HMGA2, FOS, PML, KLF7, RAD51, KIF4A, NFE2L3, MCM3, MCM4, FOSB, MCM5, MCM6, MCM2 | 2.39E-10 | 2.27E-10 |
| GOTERM_MF_DIRECT | GO:0003688~DNA replication origin binding | 8 | 4.938271605 | 6.17E-10 | CDC45, MCM7, ORC1, MCM3, MCM4, MCM5, MCM6, MCM2 | 6.10E-08 | 5.79E-08 |
| GOTERM_MF_DIRECT | GO:0005524~ATP binding | 36 | 22.22222222 | 6.60E-08 | TOP2A, DTYMK, MCM7, PIK3CD, PKMYT1, MKI67, CHTF18, AURKB, AURKA, HSP90B1, RPS6KA4, RECQL4, ORC1, PDK4, RAD54L, NEK2, MYH10, IP6K3, CCT5, RFC4, MYO10, PLK1, ATAD2, ACLY, RAD51, DDX39A, KIF4A, CDK2, MCM3, CDK1, MCM4, MCM5, MCM6, TRIP13, MET, MCM2 | 5.21E-06 | 4.95E-06 |
| GOTERM_MF_DIRECT | GO:0016887~ATPase activity | 16 | 9.87654321 | 3.33E-07 | RFC4, MCM7, ATAD2, CHTF18, HSP90B1, RECQL4, DDX39A, ORC1, MCM3, MCM4, RAD54L, MCM5, TRIP13, MCM6, MYH10, CCT5 | 2.19E-05 | 2.08E-05 |
| GOTERM_MF_DIRECT | GO:0003697~single-stranded DNA binding | 10 | 6.172839506 | 6.06E-07 | RAD51AP1, RAD51, ANXA1, CDC45, MCM7, MCM3, MCM4, MCM5, MCM6, MCM2 | 3.42E-05 | 3.25E-05 |
| GOTERM_MF_DIRECT | GO:0003678~DNA helicase activity | 8 | 4.938271605 | 8.38E-07 | RECQL4, MCM7, MCM3, RAD54L, MCM4, MCM5, MCM6, MCM2 | 4.14E-05 | 3.93E-05 |
| GOTERM_MF_DIRECT | GO:0003682~chromatin binding | 17 | 10.49382716 | 2.47E-06 | TOP2A, WDHD1, DNMT1, PCNA, CBX3, ATAD2, GMNN, FOS, KAT2B, CENPF, RAD51, CDC45, ORC1, EXO1, RAD21, CDK1, DNMT3B | 1.08E-04 | 1.03E-04 |
| GOTERM_MF_DIRECT | GO:0042826~histone deacetylase binding | 8 | 4.938271605 | 1.37E-04 | TOP2A, CDC20, KAT2B, BRMS1, GMNN, DNMT3B, KPNA2, MAGEA4 | 0.0054011 | 0.005127627 |
| GOTERM_MF_DIRECT | GO:0019901~protein kinase binding | 14 | 8.641975309 | 4.29E-04 | TFRC, RCC2, PLK1, MSN, FOXM1, CDC25B, AURKA, CCNA2, KAT2B, CCNB1, SOCS1, RACGAP1, CCNE1, E2F1 | 0.015417359 | 0.014636733 |
| GOTERM_MF_DIRECT | GO:0051015~actin filament binding | 9 | 5.555555556 | 6.93E-04 | LASP1, MYO10, ACTN1, TNNC1, CFL1, TNNC2, COTL1, MYH10, MYOM2 | 0.021481183 | 0.020393528 |
| GOTERM_MF_DIRECT | GO:0015190~L-leucine transmembrane transporter activity | 3 | 1.851851852 | 7.07E-04 | SLC7A5, SLC7A8, SLC3A2 | 0.021481183 | 0.020393528 |
| GOTERM_MF_DIRECT | GO:0003689~DNA clamp loader activity | 3 | 1.851851852 | 0.002488745 | RFC4, DSCC1, CHTF18 | 0.066286618 | 0.062930334 |
| GOTERM_MF_DIRECT | GO:0003779~actin binding | 10 | 6.172839506 | 0.002517213 | ENAH, LASP1, MYO10, CALD1, ACTN1, CFL1, TNNC2, COTL1, MSN, MYH10 | 0.066286618 | 0.062930334 |
| GOTERM_MF_DIRECT | GO:0030332~cyclin binding | 4 | 2.469135802 | 0.002978073 | CDK2, USP2, CDK1, NDC80 | 0.073521179 | 0.069798588 |
| GOTERM_MF_DIRECT | GO:0043138~3'-5' DNA helicase activity | 3 | 1.851851852 | 0.005273176 | RECQL4, MCM5, MCM2 | 0.122523796 | 0.11632006 |
| GOTERM_MF_DIRECT | GO:0019899~enzyme binding | 10 | 6.172839506 | 0.006262548 | TOP2A, CDC20, RAD51, PCNA, CBX3, COTL1, HMGA2, MSN, ENO2, MCM2 | 0.137428136 | 0.130469749 |
| GOTERM_MF_DIRECT | GO:0004029~aldehyde dehydrogenase (NAD) activity | 3 | 1.851851852 | 0.007019832 | ALDH3A1, ALDH2, ALDH1A1 | 0.142069131 | 0.134875757 |
| GOTERM_MF_DIRECT | GO:0019904~protein domain specific binding | 8 | 4.938271605 | 0.007367101 | CCNA2, CBX3, BASP1, RCC2, ACTN1, TUBB, CDK2, NEFL | 0.142069131 | 0.134875757 |
| GOTERM_MF_DIRECT | GO:0003700~transcription factor activity, sequence-specific DNA binding | 12 | 7.407407407 | 0.00788734 | TBX15, KLF7, EGR1, FOXF2, NFE2L3, MYF6, E2F1, FOSB, FOS, FOXM1, PITX1, TEAD2 | 0.142069131 | 0.134875757 |
| GOTERM_MF_DIRECT | GO:0008022~protein C-terminus binding | 7 | 4.320987654 | 0.008848141 | TOP2A, CDC20, CENPF, RAD51, PCNA, NEFL, MKI67 | 0.142069131 | 0.134875757 |
| GOTERM_MF_DIRECT | GO:0015175~neutral amino acid transmembrane transporter activity | 3 | 1.851851852 | 0.008991717 | SLC7A5, SLC7A8, SLC3A2 | 0.142069131 | 0.134875757 |
| GOTERM_MF_DIRECT | GO:0004523~RNA-DNA hybrid ribonuclease activity | 3 | 1.851851852 | 0.008991717 | FEN1, RNASEH2A, EXO1 | 0.142069131 | 0.134875757 |
| GOTERM_MF_DIRECT | GO:0008301~DNA binding, bending | 3 | 1.851851852 | 0.008991717 | TOP2A, HMGA2, HMGB3 | 0.142069131 | 0.134875757 |
| GOTERM_MF_DIRECT | GO:0016620~oxidoreductase activity, acting on the aldehyde or oxo group of donors, NAD or NADP as acceptor | 3 | 1.851851852 | 0.011180798 | ALDH3A1, ALDH2, ALDH1A1 | 0.16986212 | 0.161261507 |
| GOTERM_MF_DIRECT | GO:0000287~magnesium ion binding | 7 | 4.320987654 | 0.01482451 | TOP2A, RPS6KA4, FEN1, EPHX2, PLK1, CDK2, ENO2 | 0.209555766 | 0.198945347 |
| GOTERM_MF_DIRECT | GO:0070182~DNA polymerase binding | 3 | 1.851851852 | 0.014854586 | FANCI, RAD51, PCNA | 0.209555766 | 0.198945347 |
| GOTERM_MF_DIRECT | GO:0048256~flap endonuclease activity | 2 | 1.234567901 | 0.016939425 | FEN1, EXO1 | 0.215841063 | 0.204912402 |
| GOTERM_MF_DIRECT | GO:0097472~cyclin-dependent protein kinase activity | 2 | 1.234567901 | 0.016939425 | CDK2, CDK1 | 0.215841063 | 0.204912402 |
| GOTERM_MF_DIRECT | GO:0036461~BLOC-2 complex binding | 2 | 1.234567901 | 0.016939425 | RAB32, RAB38 | 0.215841063 | 0.204912402 |
| GOTERM_MF_DIRECT | GO:0048027~mRNA 5'-UTR binding | 3 | 1.851851852 | 0.020441737 | IGF2BP2, MYH10, CCT5 | 0.252327686 | 0.239551601 |
| GOTERM_MF_DIRECT | GO:0008475~procollagen-lysine 5-dioxygenase activity | 2 | 1.234567901 | 0.025301895 | PLOD2, PLOD1 | 0.263006539 | 0.249689752 |
| GOTERM_MF_DIRECT | GO:0018479~benzaldehyde dehydrogenase (NAD+) activity | 2 | 1.234567901 | 0.025301895 | ALDH3A1, ALDH1A1 | 0.263006539 | 0.249689752 |
| GOTERM_MF_DIRECT | GO:0035173~histone kinase activity | 2 | 1.234567901 | 0.025301895 | CDK2, CDK1 | 0.263006539 | 0.249689752 |
| GOTERM_MF_DIRECT | GO:0035651~AP-3 adaptor complex binding | 2 | 1.234567901 | 0.025301895 | RAB32, RAB38 | 0.263006539 | 0.249689752 |
| GOTERM_MF_DIRECT | GO:0015173~aromatic amino acid transmembrane transporter activity | 2 | 1.234567901 | 0.025301895 | SLC7A5, SLC3A2 | 0.263006539 | 0.249689752 |
| GOTERM_MF_DIRECT | GO:0003886~DNA (cytosine-5-)-methyltransferase activity | 2 | 1.234567901 | 0.025301895 | DNMT1, DNMT3B | 0.263006539 | 0.249689752 |
| GOTERM_MF_DIRECT | GO:0003725~double-stranded RNA binding | 4 | 2.469135802 | 0.026112143 | TFRC, ACTN1, MSN, SLC3A2 | 0.264469144 | 0.251078301 |
| GOTERM_MF_DIRECT | GO:0008134~transcription factor binding | 6 | 3.703703704 | 0.033285472 | KAT2B, FOXF2, E2F1, FOSB, HMGA2, FOS | 0.310154567 | 0.294450538 |
| GOTERM_MF_DIRECT | GO:0009008~DNA-methyltransferase activity | 2 | 1.234567901 | 0.033593669 | DNMT1, DNMT3B | 0.310154567 | 0.294450538 |
| GOTERM_MF_DIRECT | GO:1990814~DNA/DNA annealing activity | 2 | 1.234567901 | 0.033593669 | RECQL4, ANXA1 | 0.310154567 | 0.294450538 |
| GOTERM_MF_DIRECT | GO:0016538~cyclin-dependent protein serine/threonine kinase regulator activity | 3 | 1.851851852 | 0.033763662 | CCNA2, CCNB1, CCNE1 | 0.310154567 | 0.294450538 |
| GOTERM_MF_DIRECT | GO:0042803~protein homodimerization activity | 12 | 7.407407407 | 0.041404537 | TOP2A, GREM1, TBX15, CENPF, TENM3, ACOT7, TFRC, EPHX2, ACTN1, TNNC1, ECT2, PML | 0.351426819 | 0.333633056 |
| GOTERM_MF_DIRECT | GO:0004030~aldehyde dehydrogenase [NAD(P)+] activity | 2 | 1.234567901 | 0.041815343 | ALDH3A1, ALDH2 | 0.351426819 | 0.333633056 |
| GOTERM_MF_DIRECT | GO:0035650~AP-1 adaptor complex binding | 2 | 1.234567901 | 0.041815343 | RAB32, RAB38 | 0.351426819 | 0.333633056 |
| GOTERM_MF_DIRECT | GO:0035174~histone serine kinase activity | 2 | 1.234567901 | 0.041815343 | AURKB, AURKA | 0.351426819 | 0.333633056 |
| GOTERM_MF_DIRECT | GO:0004672~protein kinase activity | 8 | 4.938271605 | 0.043601603 | PLK1, CDK2, CDK1, PDK4, NEK2, PKMYT1, MET, AURKA | 0.35880486 | 0.340637525 |
| GOTERM_MF_DIRECT | GO:1990837~sequence-specific double-stranded DNA binding | 10 | 6.172839506 | 0.04841418 | TBX15, EGR1, MYF6, E2F1, FOSB, FOS, KLF15, PITX1, TEAD2, LMNB1 | 0.390277571 | 0.370516681 |
| KEGG_PATHWAY | hsa04110:Cell cycle | 21 | 12.96296296 | 4.31E-17 | PCNA, MCM7, PLK1, PKMYT1, CDC25B, CCNA2, CDC20, CCNB1, ORC6, CDC45, ORC1, CCNE1, RAD21, CDK2, MCM3, E2F1, CDK1, MCM4, MCM5, MCM6, MCM2 | 9.83E-15 | 9.71E-15 |
| KEGG_PATHWAY | hsa03030:DNA replication | 11 | 6.790123457 | 1.28E-11 | PRIM2, FEN1, RFC4, RNASEH2A, PCNA, MCM7, MCM3, MCM4, MCM5, MCM6, MCM2 | 1.46E-09 | 1.44E-09 |
| KEGG_PATHWAY | hsa04914:Progesterone-mediated oocyte maturation | 9 | 5.555555556 | 4.05E-05 | CCNA2, CCNB1, PLK1, CDK2, CDK1, PIK3CD, PKMYT1, CDC25B, AURKA | 0.003079407 | 0.003038889 |
| KEGG_PATHWAY | hsa04218:Cellular senescence | 10 | 6.172839506 | 1.47E-04 | CCNA2, CCNB1, CCNE1, CALML6, CDK2, CDK1, E2F1, RRAS2, PIK3CD, FOXM1 | 0.008394728 | 0.008284271 |
| KEGG_PATHWAY | hsa04114:Oocyte meiosis | 9 | 5.555555556 | 2.37E-04 | CDC20, CCNB1, CCNE1, CALML6, PLK1, CDK2, CDK1, PKMYT1, AURKA | 0.010787048 | 0.010645113 |
| KEGG_PATHWAY | hsa03460:Fanconi anemia pathway | 5 | 3.086419753 | 0.004676202 | FANCI, RMI2, RAD51, FANCE, FANCG | 0.177695685 | 0.175357584 |
| KEGG_PATHWAY | hsa05166:Human T-cell leukemia virus 1 infection | 9 | 5.555555556 | 0.006877424 | CCNA2, CDC20, KAT2B, EGR1, CCNE1, CDK2, E2F1, PIK3CD, FOS | 0.224007528 | 0.221060061 |
| KEGG_PATHWAY | hsa05203:Viral carcinogenesis | 8 | 4.938271605 | 0.014479709 | CCNA2, CDC20, KAT2B, CCNE1, ACTN1, CDK2, CDK1, PIK3CD | 0.412671694 | 0.407241803 |
| KEGG_PATHWAY | hsa05161:Hepatitis B | 7 | 4.320987654 | 0.016455165 | CCNA2, PCNA, CCNE1, CDK2, E2F1, PIK3CD, FOS | 0.41686418 | 0.411379125 |
| KEGG_PATHWAY | hsa03430:Mismatch repair | 3 | 1.851851852 | 0.033632822 | RFC4, PCNA, EXO1 | 0.705135351 | 0.695857254 |
| KEGG_PATHWAY | hsa05215:Prostate cancer | 5 | 3.086419753 | 0.034019688 | CCNE1, CDK2, E2F1, PIK3CD, HSP90B1 | 0.705135351 | 0.695857254 |
| KEGG_PATHWAY | hsa05206:MicroRNAs in cancer | 9 | 5.555555556 | 0.041866505 | DNMT1, SOCS1, CCNE1, E2F1, DNMT3B, HMGA2, PIK3CD, MET, CDC25B | 0.7954636 | 0.784996974 |

GO: Gene Ontology; KEGG: Kyoto Encyclopedia of Genes and Genomes
