# Supplementary material for: Prognostic model based on telomere-related genes predicts the risk of oral squamous cell carcinoma
Source: BMC Oral Health. 2023 Jul 14;23:484. doi: 10.1186/s12903-023-03157-x (PMC10347773; doi:10.1186/s12903-023-03157-x)
Supplement: Supplementary file 2 — Supplementary Material 2 [file 12903_2023_3157_MOESM2_ESM.docx]

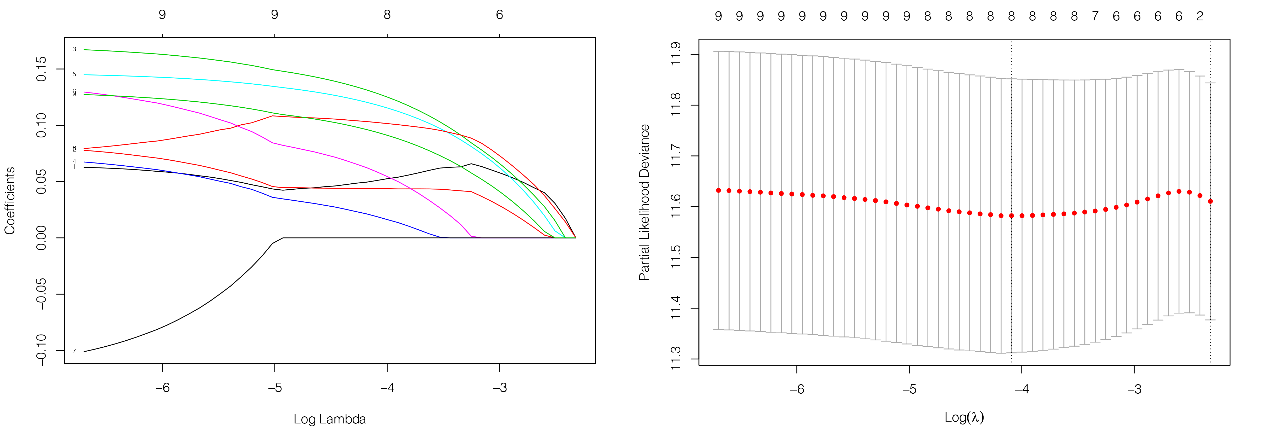
**Supplementary Figure 1 LASSO Cox analysis revealed 8 optimal DE-TRGs.** The Y-axis in the left image represented the coefficient of the variable, while the X-axis represented the value of log (lambda). The two dotted lines in the right image represented two special lambda values: lambda.min on the left and lambda.1se on the right. The lambda between these two values was considered appropriate.


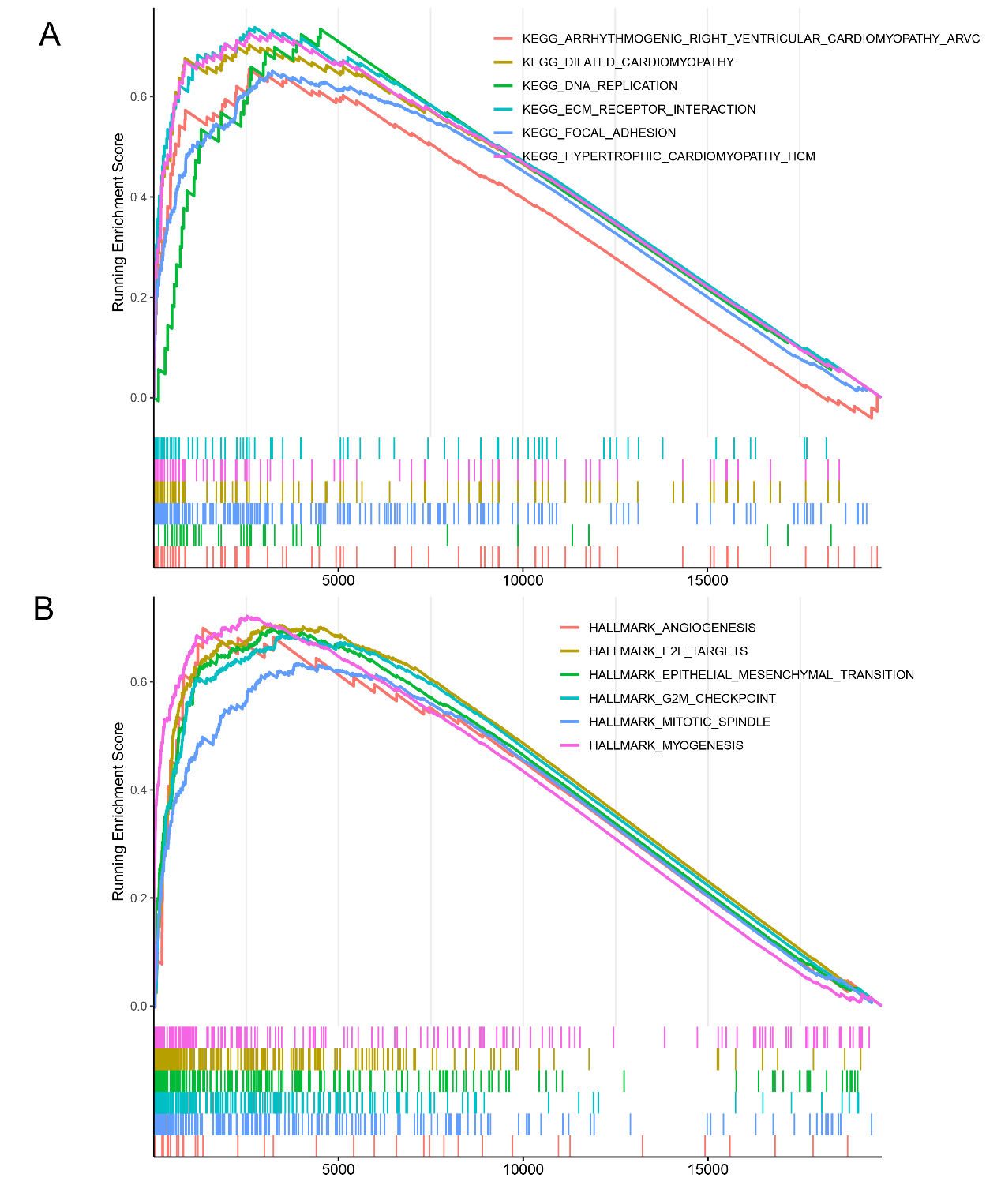


**Supplementary Figure 2 The KEGG pathway and HALLMARK gene set enrichment analysis.** A, the TOP 6 up-regulated KEGGA pathways between high-risk group and low-risk group. B, the TOP 6 up-regulated HALLMARK gene sets between high-risk group and low-risk group.

**
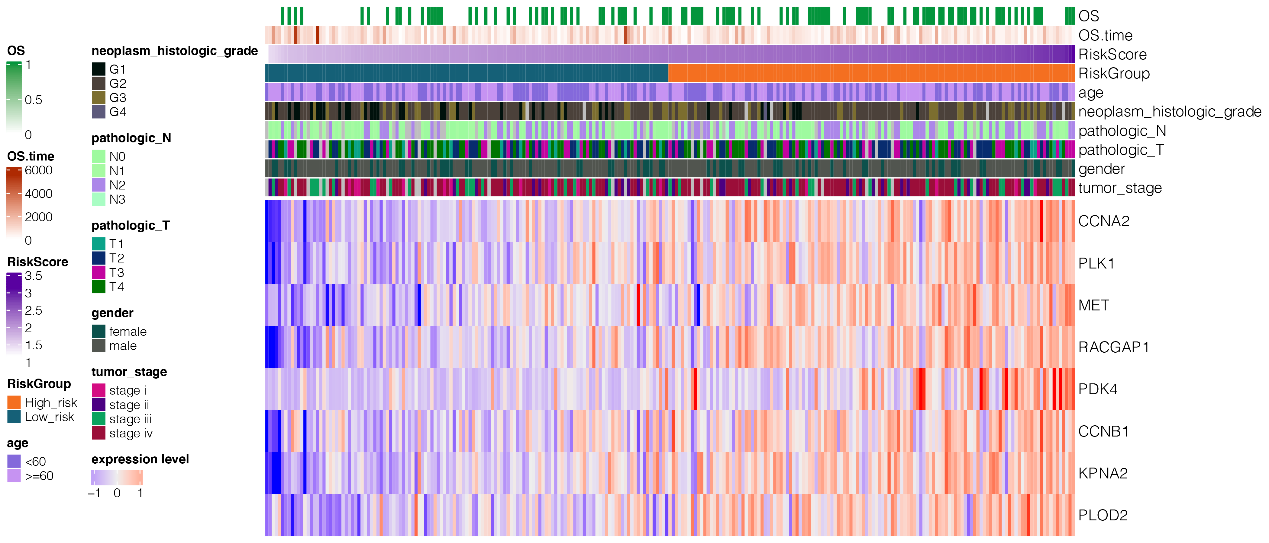
Supplementary Figure 3 The heatmap of 8 prognostic genes among different clinical groups.**
