# Supplementary figures and images for: Prognostic model based on telomere-related genes predicts the risk of oral squamous cell carcinoma
Source: BMC Oral Health. 2023 Jul 14;23:484. doi: 10.1186/s12903-023-03157-x (PMC10347773; doi:10.1186/s12903-023-03157-x)

## CCNA2

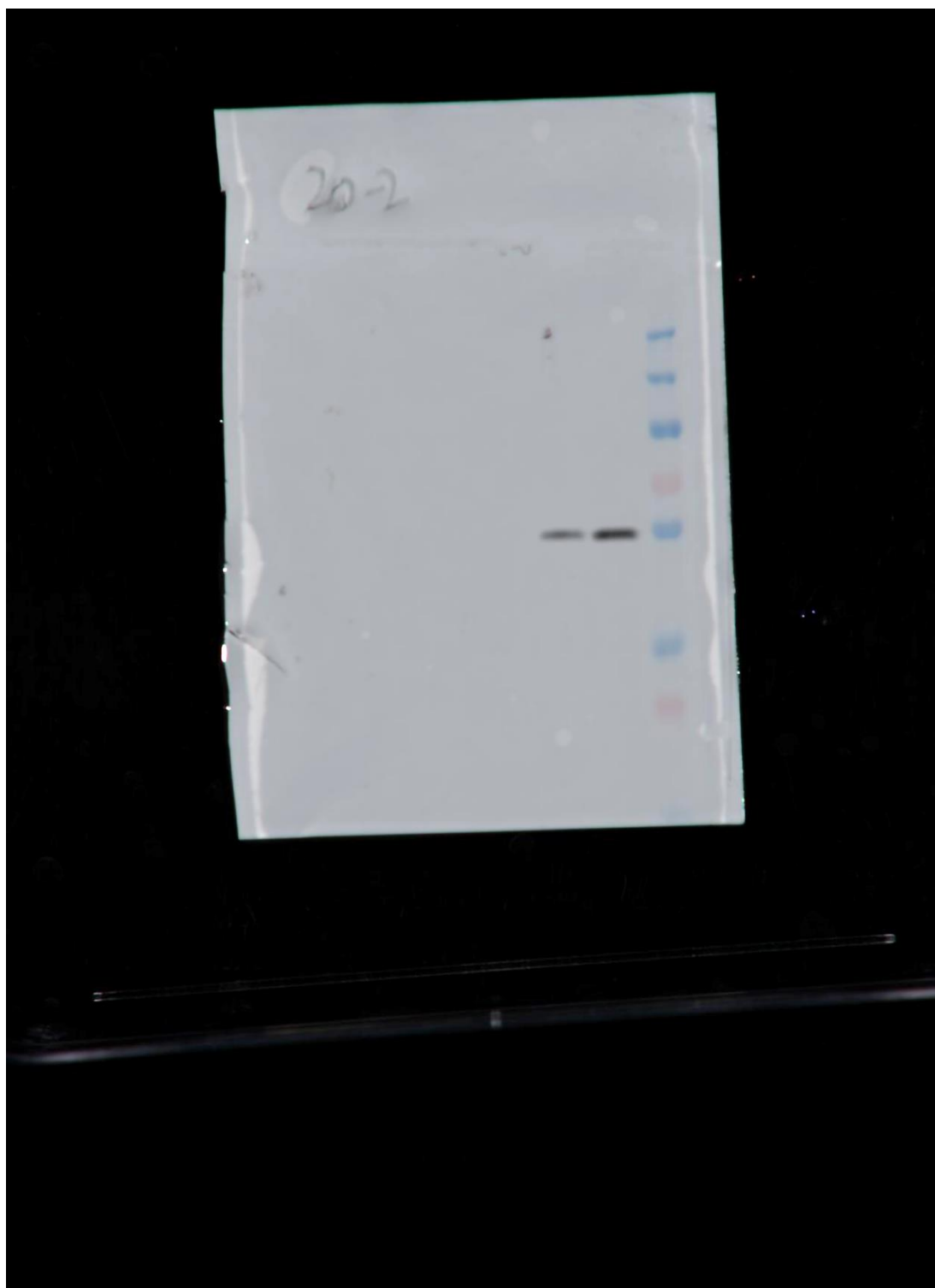

# GAPDH

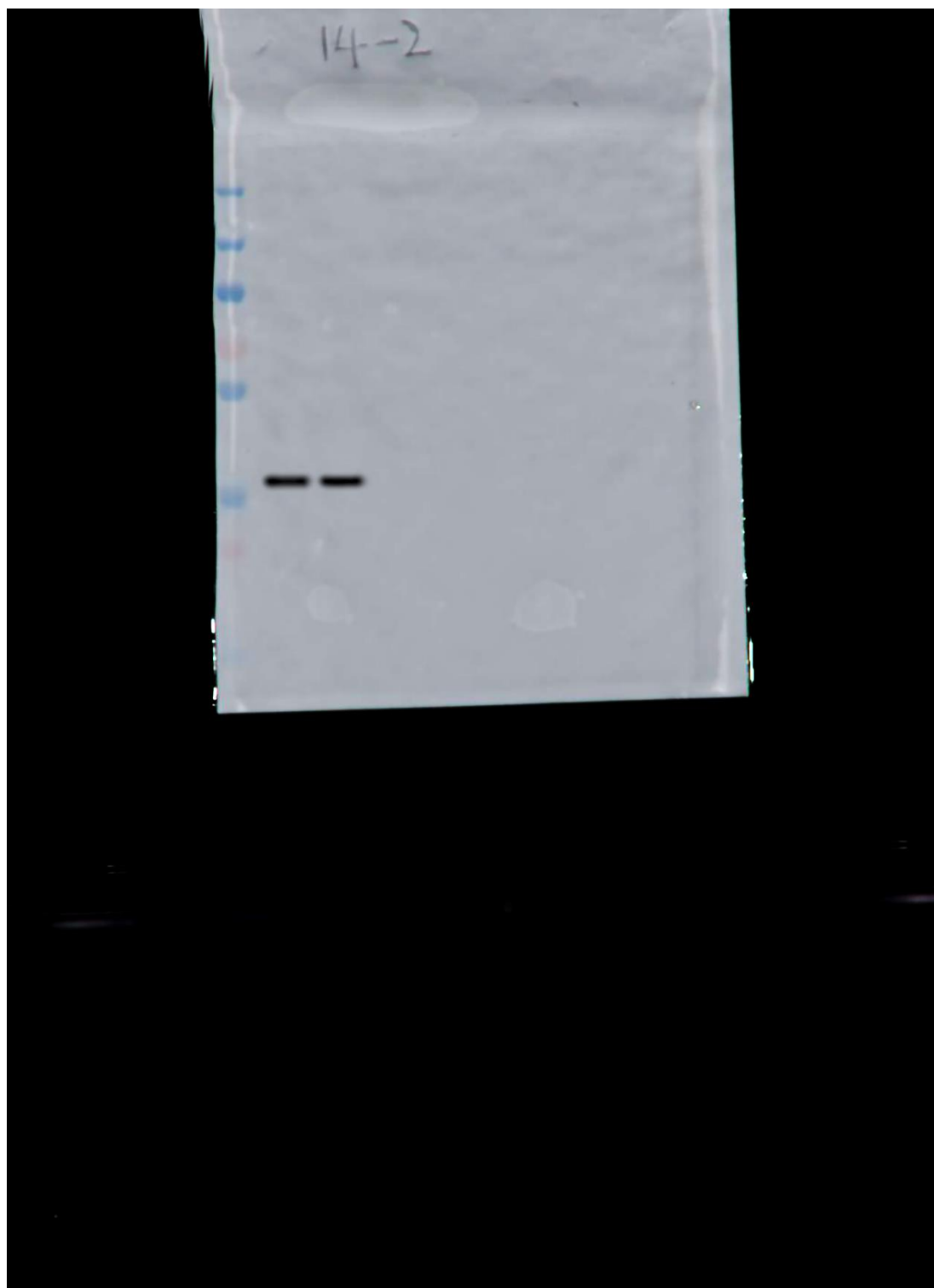

KPNA2

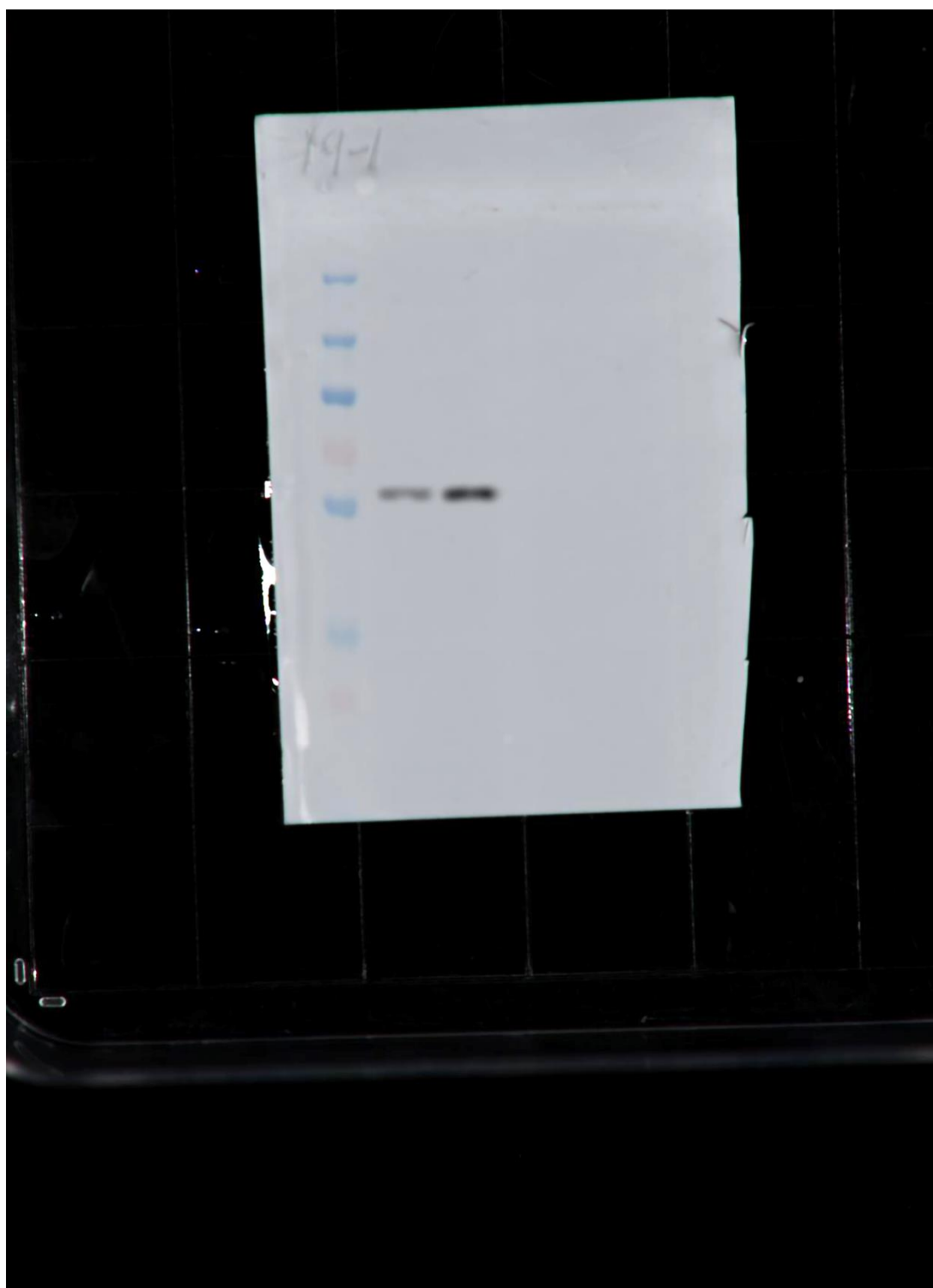

MET

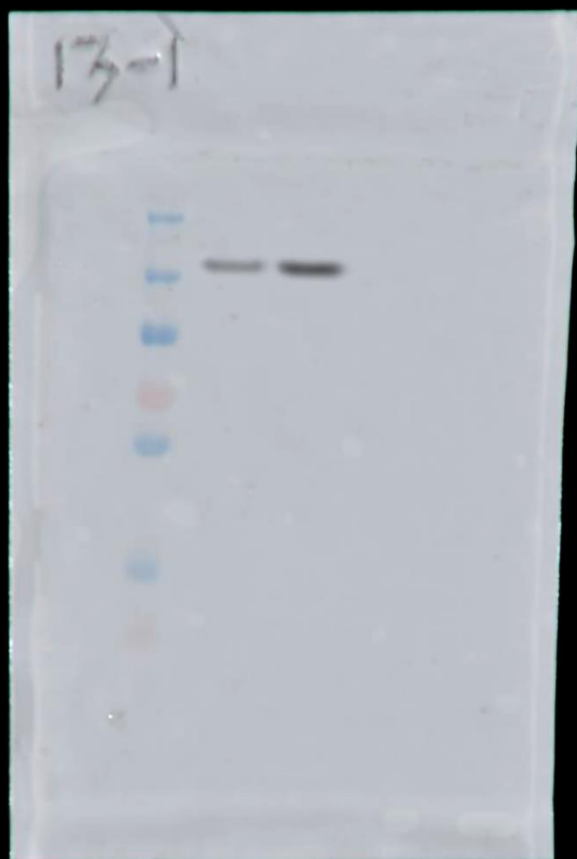

PDK4

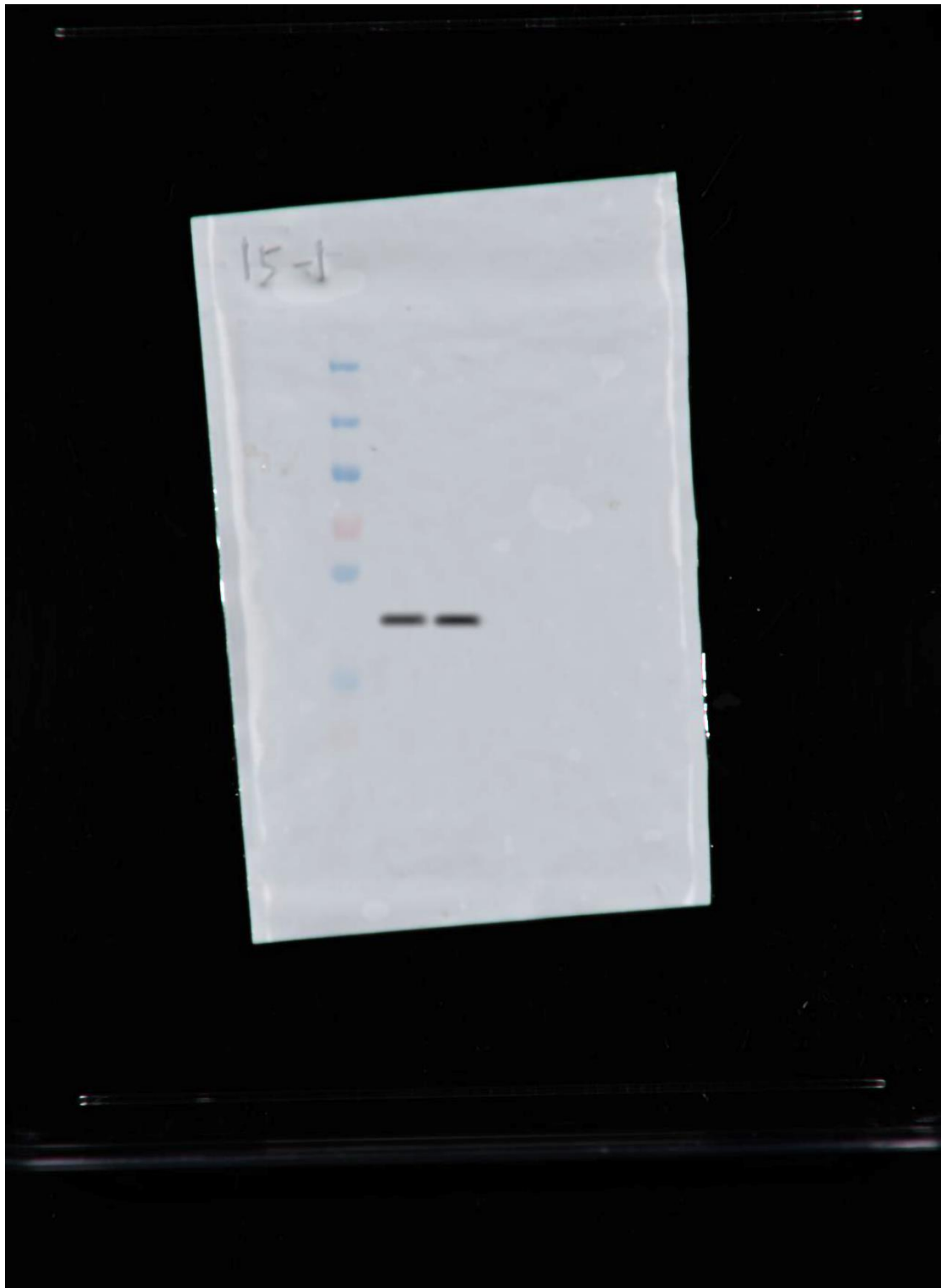

PLK1

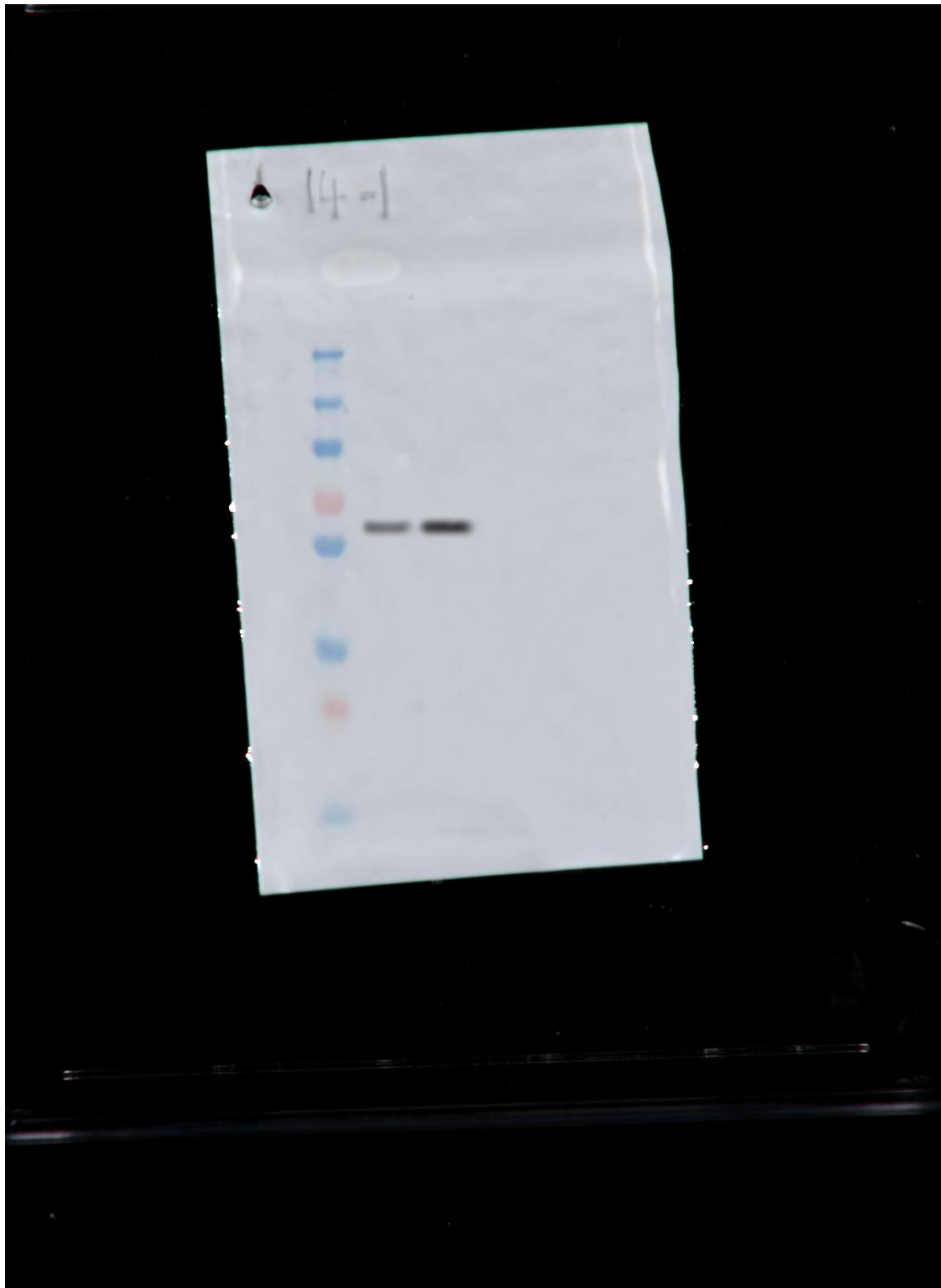

PLOD2

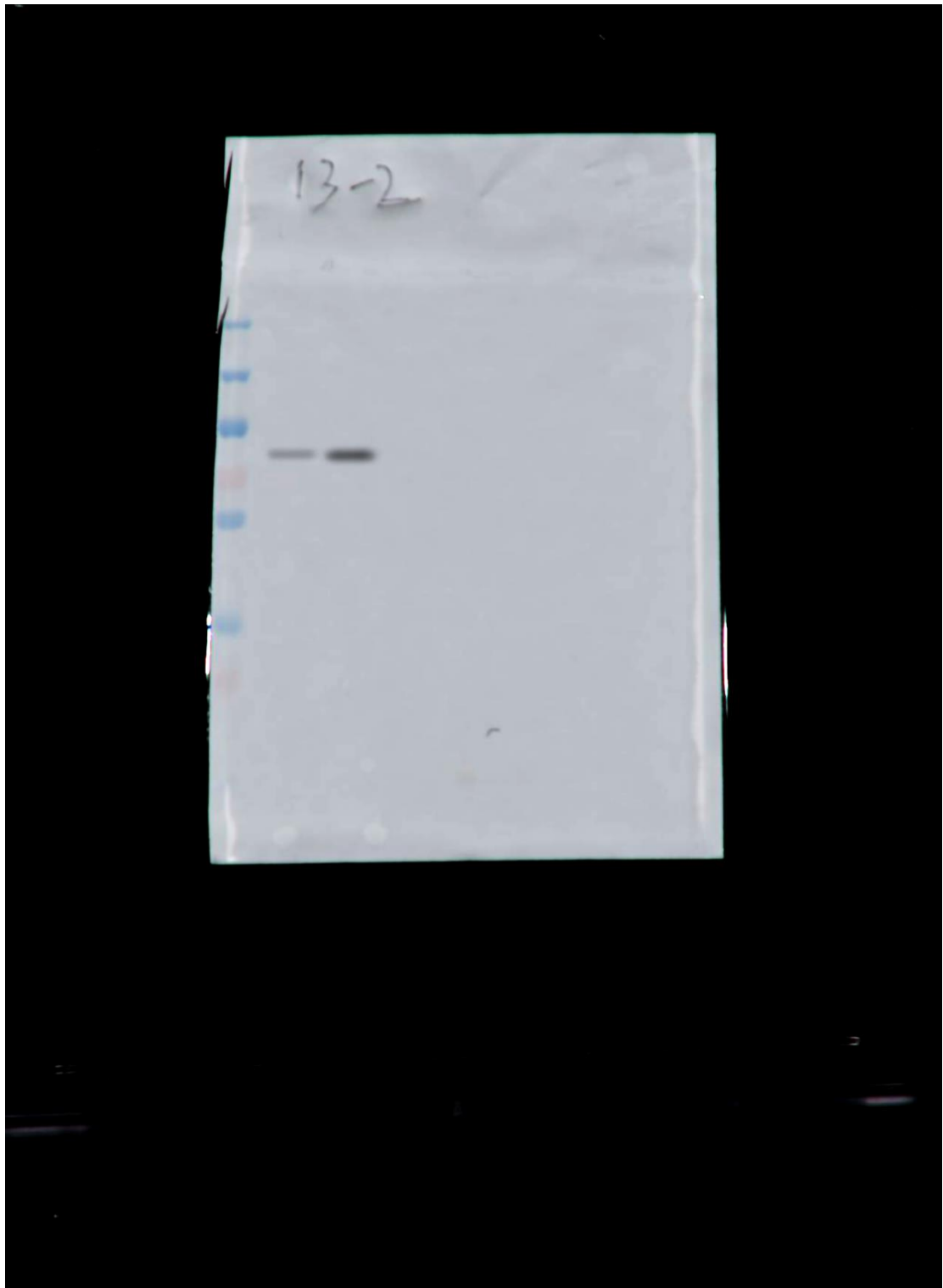

Supplement: Supplementary file 3 — Supplementary Material 3 [file 12903_2023_3157_MOESM3_ESM.pdf]
